# LncRNA INPP5F ameliorates stress‐induced hypertension via the miR‐335/Cttn axis in rostral ventrolateral medulla

**DOI:** 10.1111/cns.14142

**Published:** 2023-02-27

**Authors:** Shuai Zhang, Gaojun Chen, Xueping Wang, Lei Tong, Linping Wang, Tianfeng Liu, Liucun Zhu, Shumin Zhou, Haisheng Liu, Dongshu Du

**Affiliations:** ^1^ International Cooperation Laboratory of Molecular Medicine, Academy of Chinese Medical Sciences Zhejiang Chinese Medical University Hangzhou Zhejiang China; ^2^ School of Life Sciences Shanghai University Shanghai China; ^3^ College of Agriculture and Bioengineering Heze University Heze Shandong China; ^4^ Shaoxing Institute of Shanghai University Shaoxing Zhejiang China

**Keywords:** Cttn, lncRNA INPP5F, miR‐335, rostral ventrolateral medulla, stress‐induced hypertension

## Abstract

**Aims:**

The rostral ventrolateral medulla (RVLM) is an essential vasomotor center responsible for regulating the development of stress‐induced hypertension (SIH). Long non‐coding RNAs (lncRNAs) play critical roles in various physiopathology processes, but existing research on the functions of RVLM lncRNAs on SIH has been lacking. In this study, we investigated the roles of RVLM lncRNAs in SIH.

**Methods:**

Genome‐wide lncRNA profiles in RVLM were determined by RNA sequencing in a SIH rat model established using electric foot shocks plus noises. The hypotensive effect of lncRNA INPP5F and the underlying mechanisms of lncRNA INPP5F on SIH were explored through in vivo and in vitro experiments, such as intra‐RVLM microinjection and immunofluorescence.

**Results:**

We discovered 10,179 lncRNA transcripts, among which the lncRNA INPP5F expression level was significantly decreased in SIH rats. Overexpression of lncRNA INPP5F in RVLM dramatically reduced the blood pressure, sympathetic nerve activity, and neuronal excitability of SIH rats. LncRNA INPP5F overexpression markedly increased Cttn expression and reduced neural apoptosis by activating the PI3K‐AKT pathway, and its inhibition had opposite effects. Mechanistically, lncRNA INPP5F acted as a sponge of miR‐335, which further regulated the Cttn expression.

**Conclusion:**

LncRNA INPP5F was a key factor that inhibited SIH progression, and the identified lncRNA INPP5F/miR‐335/Cttn/PI3K‐AKT/apoptosis axis represented one of the possible mechanisms. LncRNA INPP5F could serve as a therapeutic target for SIH.

## Graphical abstract

The lncRNA INPP5F/miR‐335/Cttn/PI3K‐AKT axis modulated the apoptosis of RVLM neuronal cells, the neuronal excitability, the sympathetic nerve activity, and blood pressure, indicating that this pathway may offer a novel target for developing therapeutic strategy to treat SIH.

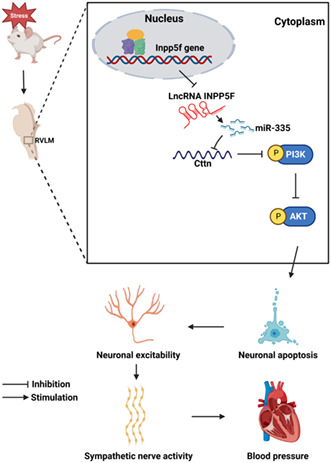

## 1 INTRODUCTION

Hypertension is a condition wherein the blood vessel has persistently elevated pressure (defined as blood pressure ≥ 140/90 mmHg). It greatly increases the risk of many other disorders, such as stroke and heart failure.^1^, ^2^ The worldwide hypertensive population continues to soar, and more than 1.56 billion adults are expected to have hypertension in 2025.^3^ Several factors, such as unhealthy diet, have been linked to hypertension risk.^4^ Studies have suggested that chronic exposure to stressors, such as job strain, could also cause hypertension.^5^, ^6^ This condition is often called stress‐induced hypertension (SIH). The autonomic nervous system and its sympathetic arm have been confirmed to exert pivotal roles in the pathogenesis of hypertension.^7^ Stress activating the sympathetic nervous system's response is believed to be involved in the development of SIH.^8^, ^9^

The rostral ventrolateral medulla (RVLM), caudal ventrolateral medulla (CVLM), nucleus tractus solitarius (NTS), and paraventricular nucleus (PVN) are important central sites that control the sympathetic outflow.^10^, ^11^ Among them, the RVLM oblongata region contains neurons that receive inputs from various sources, playing a predominant role in controlling sympathetic vasomotor tone and blood pressure.^12^ Zhang et al.^13^ concluded that PLIN2 knockdown in RVLM could block oxidative/nitrosative stress, alleviate sympathetic overdrive, and suppress SIH progression. Sigma‐1 receptor activation inhibited RVLM neuroinflammation and subsequently ameliorated sympathetic hyperactivity and blood pressure in SIH rats.^14^ Evidence revealed that NaV1.6 overexpression in RVLM mediated sympathetic activity and SIH development via glutamate regulation.^15^ Other research also indicated that the increase in sympathetic outflow by activating RVLM sympatho‐excitatory neurons in stress was responsible for generating SIH.^16^, ^17^ Thus, dysregulated gene expression in RVLM triggers the augmented sympathetic activity involved in the pathogenesis of SIH. Understanding regulatory networks of gene expression and the underlying molecular mechanisms in RVLM is necessary to slow down SIH development.

Non‐coding RNAs (ncRNAs), which are RNA molecules transcribed from the genome DNA but not encoding proteins, serve as master regulators of gene expression in diverse manners.^18^ Long non‐coding RNAs (lncRNAs) are the best‐known and largest class of regulatory ncRNAs that have a size of more than 200 bp.^19^ LncRNAs perform a wide range of functions in many complex biological and pathological processes. Several peripheral dysregulated lncRNAs have been proven to be associated with hypertension, such as lncRNA‐Ang362, lncRNA PAXIP1‐AS1, and lncRNA HOXA‐AS3.^20^, ^21^, ^22^ However, studies involving the identification of lncRNAs in the cardiovascular center and their effects on sympathetic nervous excitement and blood pressure are still in their infancy. To date, no systematic research that investigated the lncRNA profiles of RVLM participating in the pathological process of SIH has been reported.

In this work, deep RNA sequencing was performed on RVLM tissue isolated from control and SIH rats to evaluate the changes in lncRNA transcriptome systemically upon SIH. A SIH rat model was induced using electric foot shocks plus noises, and it was proposed as a plausible model for exploring the complexity of SIH.^13^, ^14^, ^15^, ^16^, ^17^ A total of 39 differentially expressed lncRNAs were uncovered based on in silico analysis upon SIH. In particular, a functionally important lncRNA, called lncRNA INPP5F, was identified. It functioned as a competing endogenous RNA (ceRNA) to target Cttn by competitively sponging miR‐335, which is required to inhibit sympathetic discharges and lower blood pressure. To our knowledge, this was the first research to build a roadmap to facilitate the discovery of functional lncRNAs for central regulation of blood pressure involved in SIH. The modulation of lncRNA INPP5F may represent a novel approach for interventional treatment of SIH.

## 2 METHODS

### 2.1 Ethics statement

All animal experiments were approved by the Animal Care Ethics Committee of Shanghai University [approval number: SYXK (HU) 2019‐0020], and they conformed to the international guidelines on the ethical use of animals.^23^, ^24^

### 2.2 Animals

**FIGURE 1:**
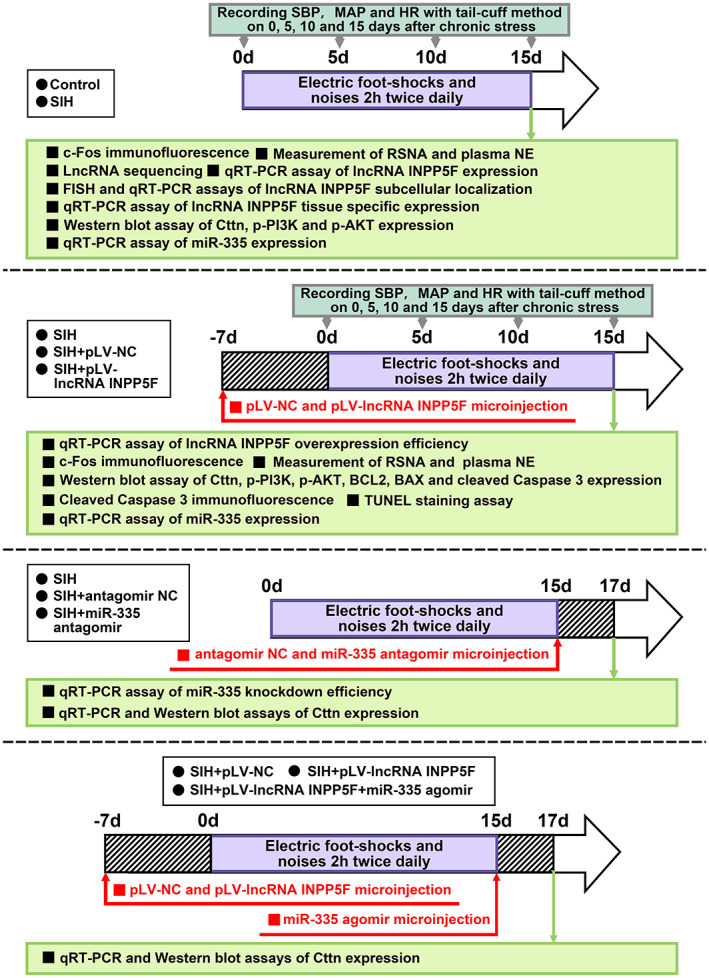
Animal experiment timeline. FISH, fluorescence in situ hybridization; HR, heart rate; MAP, mean arterial pressure; NE, norepinephrine; NC, negative control; qRT‐PCR, quantitative reverse transcription polymerase chain reaction; RSNA, renal sympathetic nerve activity; SIH, stress‐induced hypertension; SBP, systolic blood pressure; TUNEL, TdT‐mediated dUTP‐biotin nick end labeling.

Experimental male Sprague–Dawley rats (RRID: MGI: 5,651,135, *n* = 100 animals, pathogen and virus free, weight range: 230–260 g) at the age of 7 weeks were purchased from the Animal Laboratory Center of Fudan University. The rats were housed in separate cages with standard conditions (23 ± 1°C, 50%–60% humidity, and 12 h light/dark cycle) in the Laboratory Animal Center of Shanghai University and allowed free access to food and water. No exclusion criteria were pre‐determined. The animals were randomly allocated to different experimental groups. The general design of the animal experiments is depicted in Figure 1. The SIH rat model was established as previously described.^15^, ^25^ In brief, the rats were placed in a cage (22 cm × 22 cm × 28 cm) with a grid floor, and they received intermittent electric foot shocks (35–80 V for a duration of 50–100 ms), which were controlled by a computer, every 2–30 s. Meanwhile, noises with a level between 88 and 98 dB produced by a buzzer were given as the conditioned stimulus. The rats were subjected to stress stimulation for 2 h twice daily (9–11 a.m. and 3–5 p.m.) for 15 consecutive days. The control rats were placed in cages for the same period of time but not subjected to the stressful stimuli mentioned above.

### 2.3 Measurement of blood pressure and heart rate (HR)

The systolic blood pressure (SBP), mean arterial pressure (MAP), and HR of rats were measured using ALC‐NIBP non‐invasive tail‐cuff system (Alcott Biotech, China) in accordance with the manufacturer's instructions. The detection time points are illustrated in Figure 1. The rats were placed into restraining chambers for 30 min prior to recording to adapt to the measurement procedure. The whole recording process was kept in a proper environment (34 ± 0.5°C body temperature and noise‐free atmosphere). An average value of three replicate measurements for each rat was obtained.

### 2.4 Renal sympathetic nerve activity (RSNA) recording

As described previously,^25^ the RSNA in rats was recorded. In brief, a left flank incision was performed to identify and isolate the renal sympathetic nerve under isoflurane anesthesia (the rats were placed in the induction chamber, the oxygen flowmeter was adjusted to 0.8–1.5 L/min, and the isoflurane vaporizer was adjusted to 3%–5%). A pair of platinum–iridium electrodes were placed on the nerve. Subsequently, Kwik‐Sil gel (World Precision Instruments, USA) was used to cover the nerve–electrode complex, and a grass P55C preamplifier was used to amplify (×100) and filter (bandwidth: 100–3000 Hz) the nerve activity. The signal was recorded for 60 min using a Power Lab data acquisition system (RRID: SCR_001620, AD Instruments, Australia). The maximum nerve activity occurred 1–2 min after the rats were euthanized with pentobarbital sodium (≥150 mg/kg, i.p). The background noise level for the nerve activity was calculated 20–30 min after the rats were sacrificed. Baseline RSNA was taken as a percentage of maximum after the background noise was subtracted.

### 2.5 Plasma norepinephrine (NE) examination

Rat blood samples were collected by cardiac puncture using EDTA as an anticoagulant under inhalational anesthesia with isoflurane as above. They were centrifuged at 1000 ***g*** for 15 min at 4°C within 30 min after sample collection, and the supernatant was taken for detection. The level of plasma NE was tested with an ELISA kit (Cat. No. EU2565, FineTest, China) following the manufacturer's specification.

### 2.6 Total RNA extraction, lncRNA library preparation, and sequencing

The RVLM tissues were extracted by punching coronal sections according to the standard rat atlas.^26^ Total RNA was isolated from the RVLM tissues by using TRIzol reagent (Cat. No. 15596026, Invitrogen, USA) in accordance with the manufacturer's procedure. The amount and purity of RNA were measured using NanoDrop ND‐1000 (RRID: SCR_016517, Thermo Scientific, USA). The integrity of RNA was determined by the 2100 Bioanalyzer System (RRID: SCR_018043, Agilent, USA) with RNA integrity number > 7.0. A total of six high‐quality cDNA libraries were constructed, i.e., three for SIH rats and another three for control rats. Five microgram RNA per sample was utilized as input material. The Ribo‐Zero rRNA Removal Kit (Cat. No. MRZH11124, Illumina, USA) was used to deplete the ribosomal RNA. The TruSeq Stranded Total RNA Human/Mouse/Rat kit (Cat. No. 20020596, Illumina, USA) was applied to the rRNA‐depleted RNA to generate sequencing libraries. In brief, the rRNA‐depleted RNA was fragmented into small pieces by using divalent cations under high temperature. Then, Protoscript II Reverse Transcriptase and First‐strand Synthesis Mix were used to synthesize the first‐strand cDNA. The second‐strand cDNA was synthesized subsequently using *E. coli* DNA polymerase I, RNase H, and dUTP. After adenylation of the 3′ end of each strand, the indexed adapters were ligated to them. Size selection was performed by the AMPure XP System (Cat. No. A63881, Beckman, USA). The adaptor‐ligated and size‐selected products were amplified with PCR, and the PCR products were purified with AMPure XP beads (Cat. No. A63881, Beckman, USA). The library quality was assessed by the 2100 Bioanalyzer System (RRID: SCR_018043, Agilent, USA). Finally, the libraries were sequenced at Lianchuan Bio (Hangzhou, China) on the Illumina HiSeq 4000 platform (RRID: SCR_016386), and 150 bp paired‐end reads were generated.

### 2.7 Sequencing data analysis and identification of differentially expressed lncRNAs

High‐quality clean reads were obtained using Cutadapt^27^ by removing the reads that contained adaptor contamination, low‐quality bases, and undetermined bases from raw data. The clean reads were mapped to the genome of rat (ftp://ftp.ensembl.org/pub/release‐104/gtf/rattus_norvegicus/) by using Bowtie2 and Hisat2.^28^, ^29^ The mapped reads of each sample were assembled to transcripts by StringTie.^30^ Transcripts shorter than 200 bp; less than three reads coverage; and less than one exon and transcripts that overlapped with known mRNAs and other classes of RNAs, such as snRNA, snoRNA, and pseudogenes, were first discarded. The remaining transcripts were then assessed by CPC and CNCI.^31^, ^32^ Transcripts with CPC score < −1 and CNCI score < 0 were removed. The qualifying transcripts were selected and considered as lncRNAs. The expression levels of lncRNAs were measured as fragments per kilobase of exon model per million mapped fragments by using StringTie.^30^ Differential expression analysis was performed using edgeR.^33^ The *p* value was adjusted using Benjamini–Hochberg method. LncRNAs with *p*‐adjusted value <0.01 between the two groups were considered statistically significant.

### 2.8 Quantitative reverse transcription polymerase chain reaction (qRT‐PCR)

TRIzol Reagent (Cat. No. 15596026, Invitrogen, USA) was used to extract the total RNA from the RVLM tissues or B104 cells. The total RNA was reversely transcribed into cDNA by using Hifair II 1st Strand cDNA Synthesis Kit (Cat. No. 11121ES60, Yeasen, China). Meanwhile, miR‐335 was reverse transcribed individually by using Stem‐loop RT primer (5′‐GTCGTATCCAGTGCAGGGTCCGAGGTATTCGCACTGGATACGACACATTT‐3′). qRT‐PCR was then conducted on the CFX96 Touch Real‐Time PCR Detection System (RRID: SCR_018064, Bio‐Rad, USA) by using Hieff qPCR SYBR Green Master Mix (Cat. No. 11195ES03, Yeasen, China). Each reaction involved 2 μL of cDNA, 7.2 μL of H_2_O, 0.4 μL of each primer, and 10 μL of Hieff qPCR SYBR Green Master Mix. U6 was selected as an internal control in the miR‐335 qRT‐PCR assay. The relative expression levels of lncRNA INPP5F and Cttn were normalized to GAPDH. The primer sequences are listed in Table 1. The measurements were repeated three times for each rat, and the average value was taken.

**TABLE 1:** Primers used in quantitative reverse transcription polymerase chain reaction (qRT‐PCR) analysis.

Accession No.	Primer sequence (5′‐3′)	Annealing temperature (°C)	The size of production (bp)
LncRNA INPP5F	F: CCAGGTCCTCTGGGCTCTAT R: GCCCCCGGAGATAAACCAAA	60	219
LncRNA AABR07051380	F: CTGTCCTCTGGCTTCCTG R: TGATCTGCACAACCCTCAC	60	81
LncRNA AABR07060133	F: AATCCAAGTAAGCAACGA R: AAGGAGAACAGCAGTGAA	60	108
LncRNA ADGRL3	F: TGATTCAAGCACCATTCG R: CTCGGGATTAACCACAGC	60	122
LncRNA WAP1	F: TTGCTTTGTTCATATTTCTC R: TAGGCTTCTTTTGATTTGTT	60	81
LncRNA AABR07065387	F: CTCTGACTGATGGCTTGG R: AGATATGCCCTCCTATGC	60	161
LncRNA ST3GAL4	F: GCTGAGCAGGTAGTAAAT R: ACTCTGTATGGAGGTTCT	60	209
LncRNA AABR07068852	F: ATCCTCAGAACCCAACTC R: TCATACAATGCGACAAAC	60	153
LncRNA LOC102546889	F: GTCGGCTCGTTCGGTTCAT R: TCTGCCCGTCCTCATCGTC	60	121
LncRNA AABR07015078	F: TTTACGCTTTCCCTTGTCCTAG R: ATCGCTCAGCACCACCCT	60	177
Cttn	F: CAAAGGATTCGGCGGGAAGT R: TAGGCAGACGGCACCTGGAC	60	93
miR‐335	F: CGCGTCAAGAGCAATAACGAA R: AGTGCAGGGTCCGAGGTATT	60	62
U6	F: GCTTCGGCAGCACATATACTAAAAT R: CGCTTCACGAATTTGCGTGTCAT	60	94
GAPDH	F: GTCGGTGTGAACGGATTTG R: TCCCATTCTCAGCCTTGAC	60	181

Abbreviations: F, forward primer; R, reverse primer.

### 2.9 Subcellular localization of lncRNA INPP5F

The subcellular localization of lncRNA INPP5F was predicted using lncLocator and iLoc‐LncRNA.^34^, ^35^ For fluorescence in situ Hybridization (FISH), Cy3‐labeled lncRNA INPP5F and negative control (NC) probes were synthesized by GenePharma (China). Rat neuroblastoma B104 cell line (RRID: CVCL_0154) was purchased from the Shanghai Xuanya Biotechnology Co., Ltd, which has been thoroughly tested and authenticated by the supplier. B104 cells were fixed in 4% paraformaldehyde in phosphate‐buffered saline (PBS) for 15 min at room temperature and then permeabilized with 0.1% Triton X‐100 for 15 min at room temperature. Following permeabilization, the cells, in turn, were incubated with blocking buffer and saline sodium citrate (SSC) buffer for 30 min at 37°C. We diluted the FISH probes to 1 μM with sterilized DEPC water before use. Then, the cells were hybridized with Cy3‐labeled lncRNA INPP5F probe or Cy3‐labeled NC probe for 12 h at 37°C. Nuclei were counterstained by 4′,6‐diamidino‐2‐phenylindole (DAPI). Finally, images were acquired using a confocal laser scanning microscope (RRID: SCR_015963, Zeiss, Germany).

Cytoplasmic and nuclear RNAs from B104 cells were isolated and purified with the Cytoplasmic & Nuclear RNA Purification Kit (Cat. No. 21000, Norgen Biotek, USA) in accordance with the manufacturer's manual. The abundance of lncRNA INPP5F in nuclear or cytoplasmic fraction was determined by qRT‐PCR.

### 2.10 Intra‐RVLM microinjection

Intra‐RVLM microinjection was performed as described in previous study.^15^ In brief, the rats were placed in prone position, and the heads were mounted in a stereotaxic apparatus (Model 69,100, RWD Life Science, China) under inhalational anesthesia with isoflurane as described above. The skull was exposed via a midline incision. The lambda and bregma skull points were laid in the same horizontal plane. pLV‐EF1A > lncRNA INPP5F (pLV‐lncRNA INPP5F, lentiviral vector‐mediated lncRNA INPP5F overexpression, >10^9^ TU/mL) and control pLV‐EGFP:T2A:Puro‐EF1A > mCherry (pLV‐NC, >10^9^ TU/mL) plasmids were synthesized by VectorBuilder (China). MiR‐335 antagomir (1 nmol/μL), antagomir NC (1 nmol/μL), and miR‐335 agomir (1 nmol/μL) were synthesized by GenePharma (China, Table S1). They were microinjected into the bilateral RVLM (located 3.7–4.0 mm caudal to lambdoid suture, 2 mm lateral to the midline, and 8.0 mm ventral to the surface of the dura) at 1 μL/side through a glass micropipette (Figure S1). Injection sites in the area of the RVLM were confirmed by reference to the standard rat atlas of Paxinos and Watson.^26^ The time points of the microinjection are illustrated in Figure 1. After the microinjection was completed, the surgical incision was sutured and covered. No analgesic drugs were administrated after stereotaxic surgery to prevent skewing the results.

### 2.11 Cell transfection

B104 cells were cultured in high‐glucose DMEM medium (Cat. No. 10741574, Gibco, USA) supplemented with 10% fetal bovine serum (Cat. No. 11573397, Gibco, USA), and the cells were maintained in 5% CO_2_ at 37°C. At 24 h prior to transfection assay, the cells were seeded into a 6‐well plate (4 × 10^5^ cells per well), a 12‐well plate (2 × 10^5^ cells per well), or a 96‐well plate (8 × 10^3^ cells per well) and allowed to grow to 80% confluence. The cells were transfected with pLV‐lncRNA INPP5F (VectorBuilder, China) and pLV‐NC (VectorBuilder, China). LncRNA INPP5F antisense oligonucleotide (ASO, RiboBio, China, Table S1), ASO NC (RiboBio, China; the sequence was protected by a patent from RiboBio), miR‐335 agomir (GenePharma, China, Table S1), agomir NC (GenePharma, China, Table S1), miR‐335 antagomir (GenePharma, China, Table S1), and antagomir NC (GenePharma, China, Table S1) were transfected into the B104 cells with lipofectamine 8000 (Cat. No. C0533, Beyotime, China). After 24, 48, or 72 h of transfection, the cells were used for subsequent experiments.

### 2.12 Immunofluorescence

The rats were perfused through the ascending aorta with cold saline, and 4% paraformaldehyde was freshly prepared in PBS after the rats were anesthetized with pentobarbital sodium (50 mg/kg, i.p). The brain tissues were removed, post‐fixed with 4% paraformaldehyde in PBS for 12 h at room temperature, transferred to a 20% sucrose solution to dehydrate overnight at 4°C, and then transferred to a 30% sucrose solution to dehydrate overnight at 4°C. Frozen coronal sections containing RVLM were cut into 30 μm thick in a Microm HM 525 cryostat (Cat. No. 387779, Thermo Scientific, USA). B104 cells grown on glass coverslips were fixed with 4% paraformaldehyde in PBS for 30 min at room temperature. The sections or coverslips were washed in PBS, incubated with 0.3% Triton X‐100 for 30 min at room temperature, and blocked in 10% goat serum for 1 h at room temperature. The sections were incubated overnight at 4°C with the following primary antibodies: mouse monoclonal Caspase 3 antibody (1:50, RRID: AB_781826, Santa Cruz, USA), rabbit monoclonal c‐Fos (9F6) antibody (1:1000, RRID: AB_2247211, Cell Signaling Technology, USA), rabbit monoclonal NeuN antibody (1:600, RRID: AB_2532109, Abcam, USA), and mouse monoclonal tyrosine hydroxylase (F‐11) antibody (1:100, RRID: AB_628422, Santa Cruz, USA). The coverslips were incubated overnight at 4°C with the following primary antibody: mouse monoclonal Caspase 3 antibody (1:50, RRID: AB_781826, Santa Cruz, USA). Alexa Fluor 594‐conjugated AffiniPure Goat Anti‐Rabbit IgG (H + L; 1:400, RRID: AB_2338059, Jackson ImmunoResearch, USA) and FITC‐conjugated AffiniPure Goat Anti‐Mouse IgG (H + L; 1:200, RRID: AB_2338589, Jackson ImmunoResearch, USA) were used as secondary antibodies. The fluorescent signals were monitored under a confocal laser scanning microscope (RRID: SCR_015963, Zeiss, Germany).

### 2.13 Western blot

The RVLM tissues or B104 cells were lysed in RIPA buffer (Cat. No. P0013B, Beyotime, China). The concentrations of proteins were determined using the bicinchoninic acid assay kit (Cat. No. P0012, Beyotime, China). Protein was subjected to SDS/PAGE in 8%–12% gradient gel and transferred to PVDF membrane (Cat. No. IPVH00010, Millipore, USA). After being incubated with QuickBlock buffer (Cat. No. P0252, Beyotime, China) for 1 h at room temperature, the membranes were blotted with primary antibodies at 4°C overnight. The primary antibodies included rabbit polyclonal Cttn antibody (1:1000, Cat. No. A5795, ABclonal, China), mouse monoclonal Caspase 3 antibody (1:500, RRID: AB_781826, Santa Cruz, USA), rabbit polyclonal BCL2 antibody (1:1000, Cat. No. A11313, ABclonal, China), rabbit monoclonal BAX antibody (1:1000, Cat. No. CY5059, Abways, China), rabbit monoclonal phospho‐Akt (Ser473) antibody (1:2000, RRID: AB_2315049, Cell Signaling Technology, USA), rabbit polyclonal phospho‐PI3K p85 (Tyr458)/p55 (Tyr199) antibody (1:1000, RRID: AB_659940, Cell Signaling Technology, USA), and mouse monoclonal HRP‐conjugated GAPDH antibody (1:5000, RRID: AB_2737588, Proteintech, USA). The membranes were subsequently washed with PBST three times and incubated at room temperature for 1 h with secondary antibodies, including goat anti‐rabbit IgG‐HRP (1:3000, RRID: AB_2099233, Cell Signaling Technology, USA) and horse anti‐mouse IgG‐HRP (1:3000, RRID: AB_330924, Cell Signaling Technology, USA). The Super ECL detection reagent (Cat. No. 36208ES60, Yeasen, China) was utilized to detect the signals. The membranes were imaged by an automatic chemiluminescence image analysis system (Model 5200, Tanon Science & Technology, China). GAPDH was used as a loading control to normalize the data.

### 2.14 TdT‐mediated dUTP‐biotin nick end‐labeling (TUNEL) assay

The YF 488 TUNEL apoptosis detection kit (Cat. No. T6013L, UElandy, China) was used to detect cell apoptosis in accordance with the manufacturer's specifications. Frozen RVLM sections were fixed with 4% paraformaldehyde in PBS for 30 min at room temperature, washed twice with PBS, and incubated with 20 μg/mL proteinase K in PBS for 20 min at room temperature. The B104 cells grown on glass coverslips were fixed with 4% paraformaldehyde in PBS for 30 min at room temperature. The coverslips were washed twice with PBS and incubated with 0.3% Triton X‐100 for 20 min at room temperature. The sections or coverslips were then incubated with TUNEL equilibration buffer for 5 min at room temperature, followed by incubation with TUNEL reaction buffer (TdT enzyme added) for 1–2 h at 37°C in the dark. Finally, the samples were stained with DAPI for 30 min at room temperature in the dark. The signals were detected by a confocal laser scanning microscope (RRID: SCR_015963, Zeiss, Germany).

### 2.15 Calcein/propidium iodide (PI) assay

Cell viability was detected using Calcein/PI Cell Viability/Cytotoxicity Assay Kit (Cat. No. C2015S, Beyotime, China) following the manufacturer's directions. In brief, B104 cells were seeded into six‐well plates and transfected with lncRNA INPP5F ASO (RiboBio, China, Table S1) or ASO NC (RiboBio, China; the sequence was protected by a patent from RiboBio) for 48 h. The cells were then washed with PBS and incubated with Calcein AM/PI detection buffer for 30 min at 37°C in the dark. Finally, they were observed under a confocal laser scanning microscope (RRID: SCR_015963, Ziess, Germany).

### 2.16 Cell counting kit‐8 (CCK‐8) assay

CCK‐8 assay was conducted using a commercial kit (Cat. No. B34302, Bimake, China), following the procedure provided by the manufacturer. In brief, B104 cells transfected with lncRNA INPP5F ASO (RiboBio, China, Table S1) or ASO NC (RiboBio, China; the sequence was protected by a patent from RiboBio) were plated in 96‐well plates for 24, 48, and 72 h. Then, a CCK‐8 solution was added into each well of the plate and incubated together for 1 h. Absorbance at 450 nm was evaluated using a LabServ K3 microplate reader (Cat. No. 117123002, Thermo Scientific, USA).

### 2.17 Flow cytometry

The apoptosis of B104 cells was examined using the Annexin V‐Alexa Fluor 647/PI apoptosis detection kit (Cat. No. 40304ES60, Yeasen, China) following the manufacturer's instructions. In brief, B104 cells were cultured in six‐well plates and transfected with lncRNA INPP5F ASO (RiboBio, China, Table S1) or ASO NC (RiboBio, China; the sequence was protected by a patent from RiboBio). After 48 h, the cells were collected, washed twice in cold PBS, and resuspended with 1× binding buffer. Then, they were incubated with Alexa Fluor 647‐Annexin V and PI for 15 min at room temperature in the dark. After 1× binding buffer was added into each well, the apoptotic cells were quantified using a flow cytometer (Cat. No. C00445, Beckman, USA).

### 2.18 Dual‐luciferase reporter assay

The wild‐type (WT) and mutant (MUT)‐binding sites of miR‐335 in lncRNA INPP5F fragment or Cttn 3′ untranslated region (UTR) were subcloned into pmirGLO dual‐luciferase miRNA target expression vector to construct lncRNA INPP5F‐WT/MUT or Cttn‐WT‐3′ UTR/MUT‐3′ UTR. The pmirGLO plasmids were co‐transfected with miR‐335 agomir (GenePharma, China, Table S1) or agomir NC (GenePharma, China, Table S1) into B104 cells. After 48 h of transfection, the luciferase activity was tested by the dual‐luciferase reporter assay system (Cat. No. 11402ES60, Yeasen, China) in accordance with the manufacturer's instructions.

### 2.19 Biotinylated RNA pull‐down assay

B104 cells with lncRNA INPP5F overexpression were transfected with biotinylated miR‐335 agomir (GenePharma, China, Table S1) or biotinylated agomir NC (GenePharma, China, Table S1). The cells were lysed and incubated with streptavidin magnetic beads (Cat. No. 88816, Thermo Scientific, USA), followed by qRT‐PCR assay.

### 2.20 Statistical analysis

Data were assessed with version 9.1 GraphPad Prism software and expressed as the mean ± standard error of the mean (SEM). Samples sizes used were similar to those used in our previous studies.^15^, ^17^, ^25^ Shapiro–Wilk method was used to evaluate the normality of the data. The data conformed to a normal distribution. Two‐tailed unpaired Student's *t*‐test was conducted to assess the significant differences between two groups, and one‐way ANOVA with subsequent post hoc Bonferroni test was performed to compare multiple groups. *p* < 0.05 was defined as statistically significant. The full statistical reports are shown in Table S2.

## 3 RESULTS

### 3.1 LncRNA INPP5F expression was downregulated in RVLM of SIH rats

**FIGURE 2:**
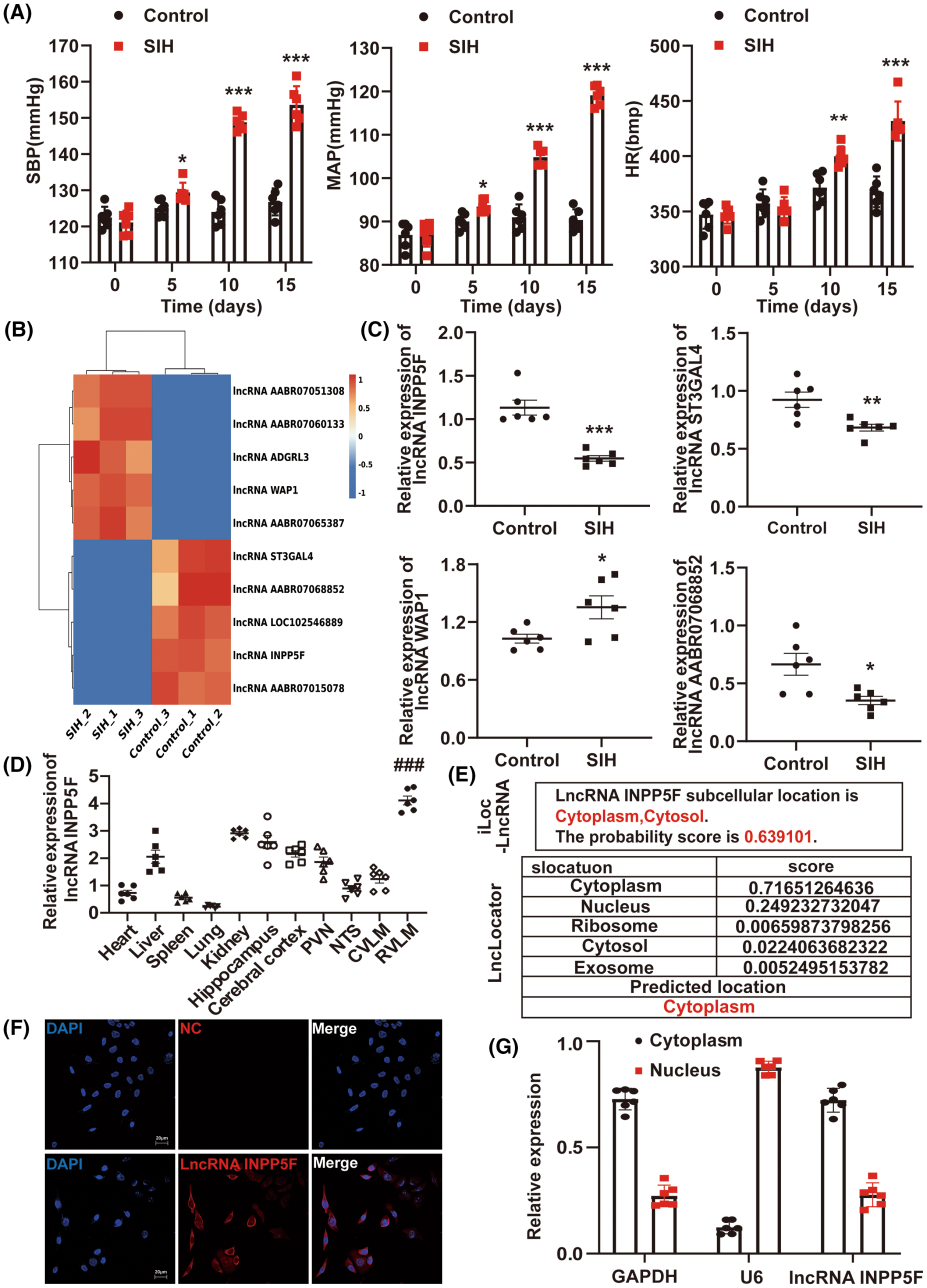
LncRNA INPP5F was downexpressed in RVLM after 15 days of stress exposure in rats. (A) Chronic stress increased the levels of SBP, MAP, and HR in rats. (B) Cluster analysis of the top five most increased and decreased lncRNAs identified by RNA sequencing in the control and SIH rats. (C) Relative expression of the four indicated lncRNAs listed in (B) measured by qRT‐PCR. (D) Relative expression of lncRNA INPP5F in 11 tissues of SIH rats, as determined by qRT‐PCR. (E) Bioinformatics prediction of the subcellular location of lncRNA INPP5F by lncLocator and iLoc‐LncRNA software. (F) Subcellular localization of lncRNA INPP5F in B104 cells using FISH. NC means Cy3‐labeled negative control probe. (G) qRT‐PCR was used to measure the expression of lncRNA INPP5F in either the nucleus or cytoplasm. Data were presented as mean ± SEM. Statistical significance was determined by two‐tailed unpaired Student's *t*‐test (A, C) and one‐way ANOVA, followed by post hoc Bonferroni test (D). *n* = 6 rats per group (A, C, D). *n* = 6 of independent cell culture preparations (G). *p < 0.05, **p < 0.01, and ***p < 0.001 versus control group. ^###^ *p* < 0.001 versus heart group. DAPI, 4′,6‐diamidino‐2‐phenylindole; FISH, fluorescence in situ hybridization; HR, heart rate; MAP, mean arterial pressure; NC, negative control; qRT‐PCR, quantitative reverse transcription polymerase chain reaction; RVLM, rostral ventrolateral medulla; SBP, systolic blood pressure; SEM, standard error of the mean; SIH, stress‐induced hypertension.

After 15 days of stress, changes in blood pressure, HR, sympathetic nerve activity, and neuronal excitability were examined between the two groups. The results suggested that the SIH rats displayed observably higher SBP, MAP, HR, RSNA, plasma NE, and percentage of c‐Fos‐positive tyrosine hydroxylase+ (TH+) neurons (Figure 2A and Figure S2A–C). The weight of SIH rats was lower than that in the control rats but markedly increased after the 12th day of stress (Figure S3). This result was observed to be consistent with our previous study.^17^ These data revealed that the SIH rat model was established successfully. RNA sequencing was used to assess the lncRNA expression profiles in RVLM tissues of the control and SIH rats. A total of 524,098,064 raw reads (265,059,924 for SIH rats and 259,038,140 for control rats) were generated. After the low‐quality reads were discarded, 501,346,052 clean reads (254,254,380 for SIH rats and 247,091,672 for control rats) were detected. We aligned the clean reads to the rat reference genome, and the aligning rates were about 76.2% and 75.5% in the SIH and control groups, respectively. A total of 10,179 lncRNAs were identified and used for subsequent analyses. With the *p*‐adjusted value <0.01 as the significance threshold, 39 differentially expressed lncRNAs in the SIH and control rats were detected. Through expression intensity sorting within SIH and control groups, the five mostly increased and decreased lncRNAs in SIH rats as compared to that in control rats are shown in Figure 2B. Next, the qRT‐PCR was carried out to confirm the expression of these 10 mostly changed lncRNAs in RVLM between the two groups, 4 of which exhibited differential expression and agreed with the lncRNA sequencing results (Figure 2C). Some data do not correspond to the lncRNA sequencing results (Figure S4), which may be due to the biological differences between samples. Among them, we found that lncRNA INPP5F expression was consistently and significantly decreased in SIH rats as compared to that in matched controls (Figure 2C). Moreover, the tissue‐specific expression of lncRNA INPP5F was determined using qRT‐PCR. LncRNA INPP5F was abundantly expressed in RVLM tissue of both SIH and control rats (Figure 2D and Figure S5). Thus, we focused on the expression and role of lncRNA INPP5F in SIH progression in this study. The subcellular location of lncRNA INPP5F was evaluated. The bioinformatics prediction, FISH, and qRT‐PCR results showed that lncRNA INPP5F was mainly distributed in the cytoplasm, manifesting that it might affect gene expression by binding miRNAs (Figure 2E–G).^36^

### 3.2 LncRNA INPP5F overexpression suppressed SIH progression

**FIGURE 3:**
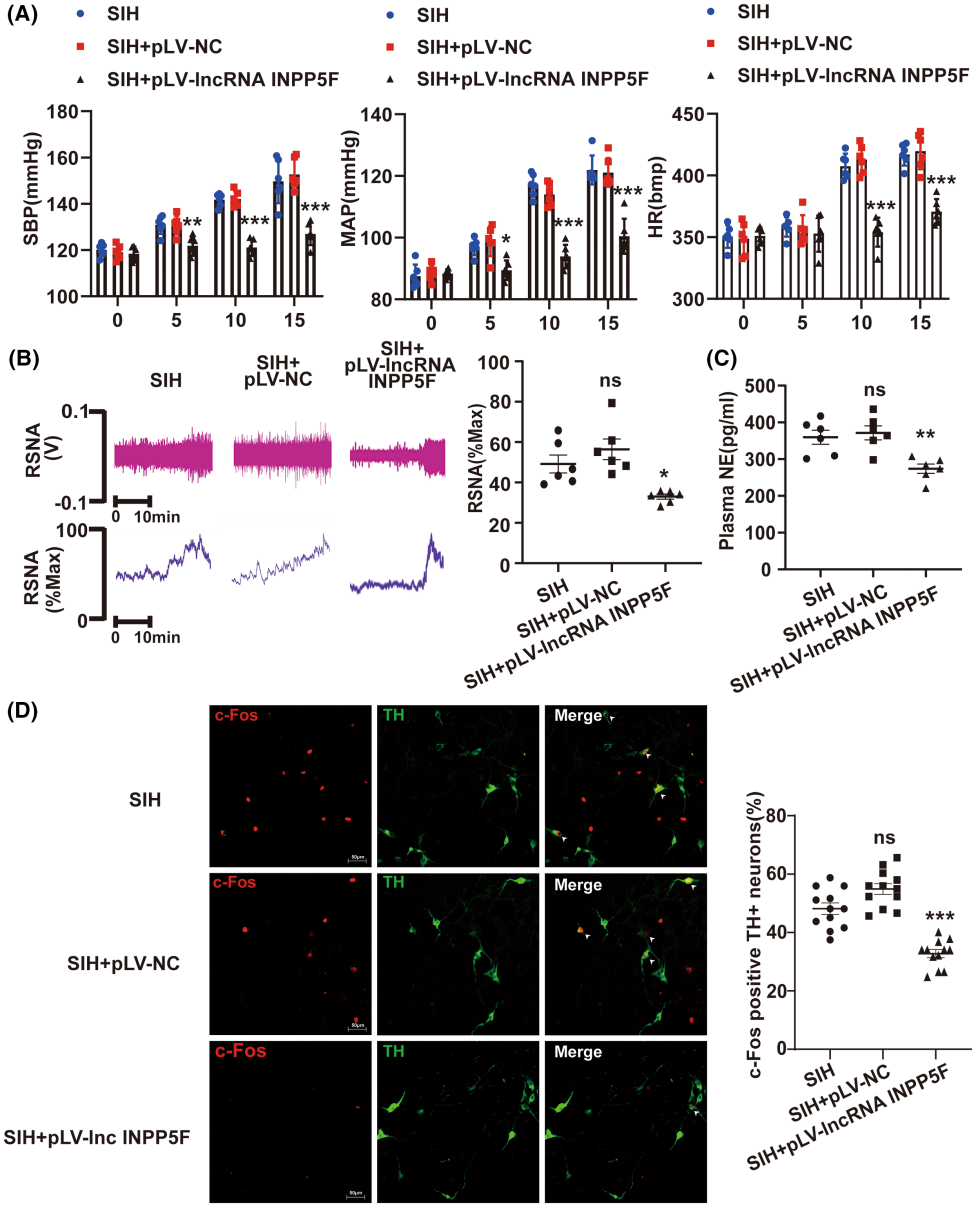
Upregulated lncRNA INPP5F expression in RVLM contributed to improve SIH. (A) Tail‐cuff method was employed to record SBP, MAP, and HR in rats after lncRNA INPP5F overexpression. (B, C) RSNA recording and plasma NE ELISA test of SIH rats microinjected with pLV‐lncRNA INPP5F to evaluate sympathetic nerve activity. (D) Representative immunofluorescent image of co‐localization of c‐fos with TH and percentage of c‐Fos‐positive TH+ neurons in RVLM. Data were presented as mean ± SEM. Statistical significance was determined by one‐way ANOVA, followed by post hoc Bonferroni test (A–D). *n* = 6 rats per group (A–C). *n* = 12 slices from 6 rats, two slices per rat (D). *p < 0.05, **p < 0.01, and ***p < 0.001 versus SIH group. ns means non‐significant versus SIH group. HR, heart rate; MAP, mean arterial pressure; NC, negative control; NE, norepinephrine; RVLM, rostral ventrolateral medulla; RSNA, renal sympathetic nerve activity; SBP, systolic blood pressure; SEM, standard error of the mean; SIH, stress‐induced hypertension; TH, tyrosine hydroxylase.

The RVLM of SIH rats was bilaterally microinjected with pLV‐lncRNA INPP5F (VectorBuilder, China) or pLV‐NC (VectorBuilder, China) plasmid 1 week prior to stress application. As shown in Figure S6A, the pLV‐lncRNA INPP5F plasmid obviously increased the lncRNA INPP5F expression. The SBP, MAP, and HR levels of SIH rats were remarkably attenuated by lncRNA INPP5F overexpression (Figure 3A). Next, the results of RSNA recording and plasma NE ELISA test showed that the RSNA and plasma NE values in the SIH + pLV‐lncRNA INPP5F rats were robustly decreased compared with those in the SIH and SIH + pLV‐NC rats (Figure 3B,C). Furthermore, c‐Fos protein was determined by immunofluorescence assay. The findings suggested that lncRNA INPP5F upregulation significantly reduced the proportion of c‐Fos‐positive TH+ neurons in RVLM of SIH rats (Figure 3D). All these data indicated that the increased expression of lncRNA INPP5F restored neuronal excitability, inhibited sympathetic nerve activity, and participated in blood pressure recovery.

### 3.3 LncRNA INPP5F functioned as a positive regulator of Cttn and restrained apoptosis by activating the PI3K‐AKT pathway

**FIGURE 4:**
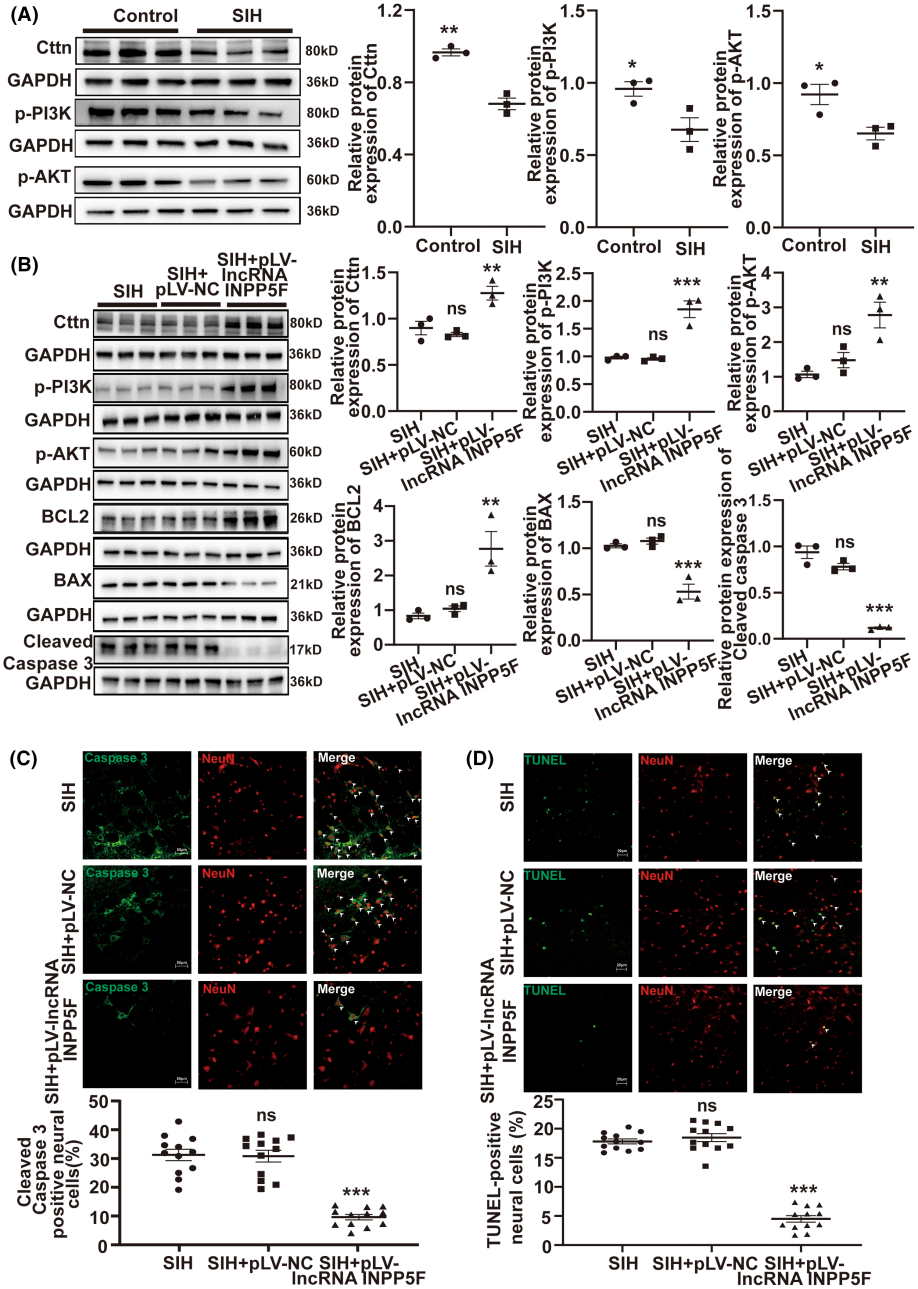
LncRNA INPP5F attenuated apoptosis in SIH via activating Cttn/PI3K‐AKT signal pathway. (A) Western blot analysis of Cttn, p‐PI3K, and p‐AKT expression in RVLM of SIH and control rats. (B–D) After microinjection of pLV‐lncRNA INPP5F plasmid in RVLM of SIH rats, Cttn, p‐PI3K, p‐AKT, BCL2, BAX, and cleaved Caspase 3 protein levels were detected by Western blot, cleaved Caspase 3 protein was determined by immunofluorescence, and apoptosis level was tested by TUNEL staining. Data were presented as mean ± SEM. Statistical significance was determined by two‐tailed unpaired Student's *t*‐test (A) and one‐way ANOVA, followed by post hoc Bonferroni test (B–D). *n* = 3 rats per group (A, B). *n* = 12 slices from 6 rats, two slices per rat (C, D). *p < 0.05, **p < 0.01, and ***p < 0.001 versus SIH group. ns means non‐significant versus SIH group. DAPI, 4′,6‐diamidino‐2‐phenylindole; NC, negative control; RVLM, rostral ventrolateral medulla; SEM, standard error of the mean; SIH, stress‐induced hypertension; TUNEL, TdT‐mediated dUTP‐biotin nick end labeling.

**FIGURE 5:**
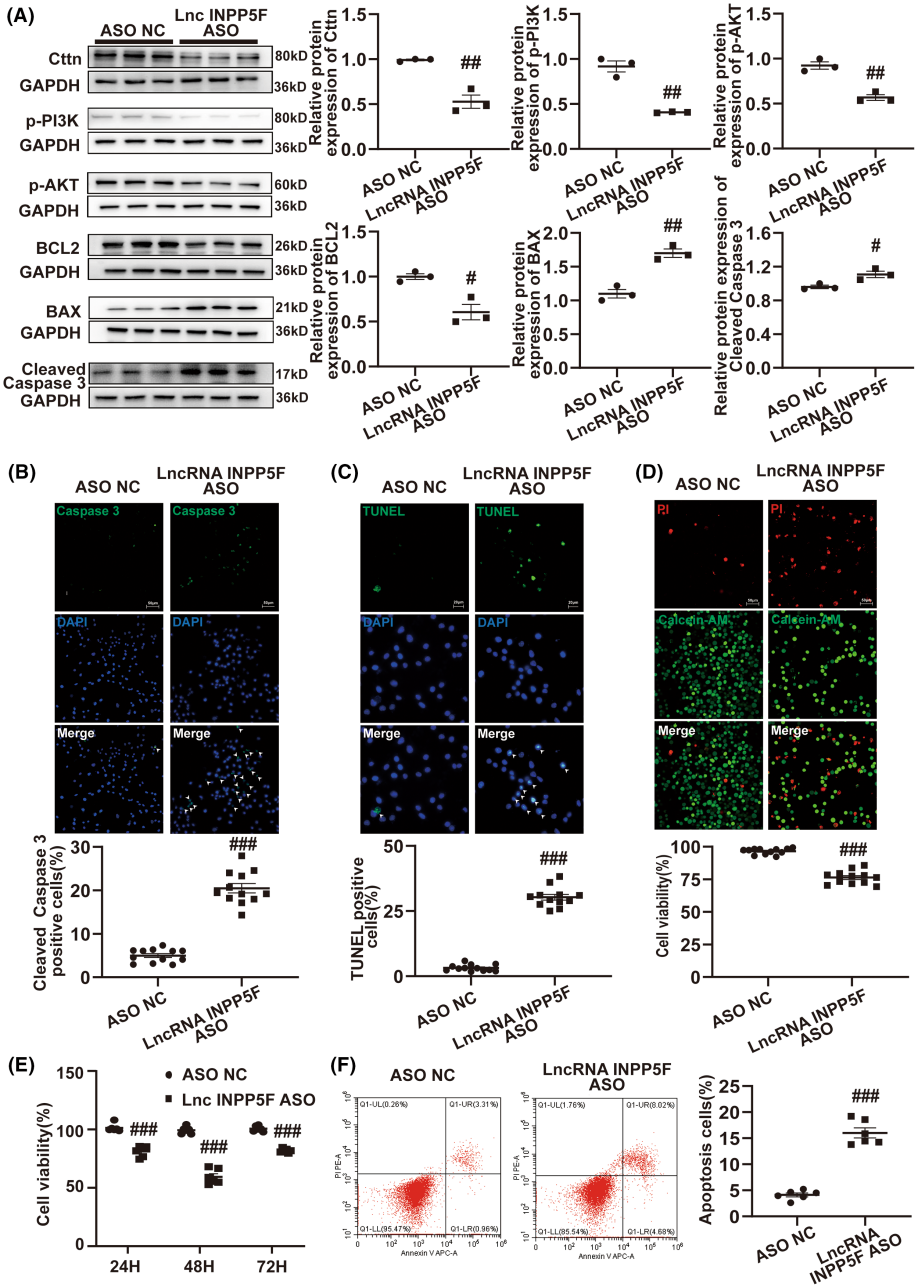
LncRNA INPP5F knockdown induced apoptosis by regulating Cttn/PI3K‐AKT signal pathway in B104 cells. (A–C) After transfection of lncRNA INPP5F ASO for 48 h in B104 cells, Cttn, p‐PI3K, p‐AKT, BCL2, BAX, and cleaved Caspase 3 protein levels were assessed by Western blot, cleaved Caspase 3 protein was explored by immunofluorescence, and apoptosis level was investigated by TUNEL staining. (D) Cell viability was surveyed by Calcein/PI staining in B104 cells after transfection of lncRNA INPP5F ASO for 48 h. (E) CCK‐8 assay for examining cell viability in B104 cells after transfection of lncRNA INPP5F ASO for 24, 48, and 72 h. (F) Apoptosis rate was evaluated by flow cytometry in B104 cells after transfection of lncRNA INPP5F ASO for 48 h. Data were presented as mean ± SEM. Statistical significance was determined by two‐tailed unpaired Student's *t*‐test (A–F). *n* = 3 of independent cell culture preparations (A). *n* = 6 of independent cell culture preparations (E, F). *n* = 12 slices from 6 of independent cell culture preparations, 2 slices per independent cell culture preparation (B–D). ^#^ *p* < 0.05, ^##^ *p* < 0.01, and ^###^ *p* < 0.001 versus ASO NC group. ASO, antisense oligonucleotide; CCK‐8, cell counting kit‐8; DAPI, 4′,6‐diamidino‐2‐phenylindole; NC, negative control; PI, propidium iodide; SEM, standard error of the mean; TUNEL, TdT‐mediated dUTP‐biotin nick end labeling.

Neuronal apoptosis in RVLM contributes to sympathetic overactivity and increases the risk of hypertension.^25^, ^37^ Moreover, Cttn strongly affects apoptosis.^38^, ^39^ The PI3K‐AKT signal pathway has been proven to play an important role in apoptosis.^40^, ^41^ Cttn is associated with the PI3K‐AKT pathway,^42^, ^43^ which participates in apoptosis.^43^, ^44^ In the present study, the expression levels of Cttn, p‐PI3K, and p‐AKT in RVLM were detected by Western blot in SIH and control rats. We observed that compared with the control rats, Cttn, p‐PI3K, and p‐AKT protein expression levels were markedly decreased in the SIH rats (Figure 4A). Western blot was also conducted to evaluate the expression of Cttn, p‐PI3K, p‐AKT, BCL2, BAX, and cleaved Caspase 3 in RVLM of SIH rats following pLV‐lncRNA INPP5F plasmid microinjection. As shown in Figure 4B, the lncRNA INPP5F upregulation in SIH rats significantly increased Cttn, p‐PI3K, p‐AKT, and BCL2 expression and reduced that of BAX and cleaved Caspase 3. Immunofluorescence assay proved that lncRNA INPP5F overexpression in RVLM remarkably lowered the percentage of cleaved Caspase 3‐positive neural cells in SIH rats (Figure 4C). We performed TUNEL staining to further test the cell apoptosis level. The group overexpressed with lncRNA INPP5F had significantly decreased number of TUNEL‐positive neural cells (Figure 4D). LncRNA INPP5F ASO and ASO NC were transfected into B104 cells for in vitro study. The knockdown efficiency of lncRNA INPP5F ASO is shown in Figure S6B. Western blot indicated that lncRNA INPP5F knockdown significantly reduced the expression levels of Cttn, p‐PI3K, p‐AKT, and BCL2 and increased those of BAX and cleaved Caspase 3 (Figure 5A). Compared with the ASO NC group, the group treated with lncRNA INPP5F ASO raised the proportions of cleaved Caspase 3‐positive cells and TUNEL‐positive cells uncovered by immunofluorescence and TUNEL staining (Figure 5B,C). Furthermore, the Calcein/PI, CCK‐8, and flow cytometry experiments revealed that lncRNA INPP5F silencing inhibited cell viability (Figure 5D,E) and induced apoptosis (Figure 5F). These above results implicated that lncRNA INPP5F repressed cell apoptosis by activating the Cttn/PI3K‐AKT pathway in RVLM to lessen sympathetic outflow, further against SIH progression.

### 3.4 LncRNA INPP5F acted as a sponge of miR‐335

**FIGURE 6:**
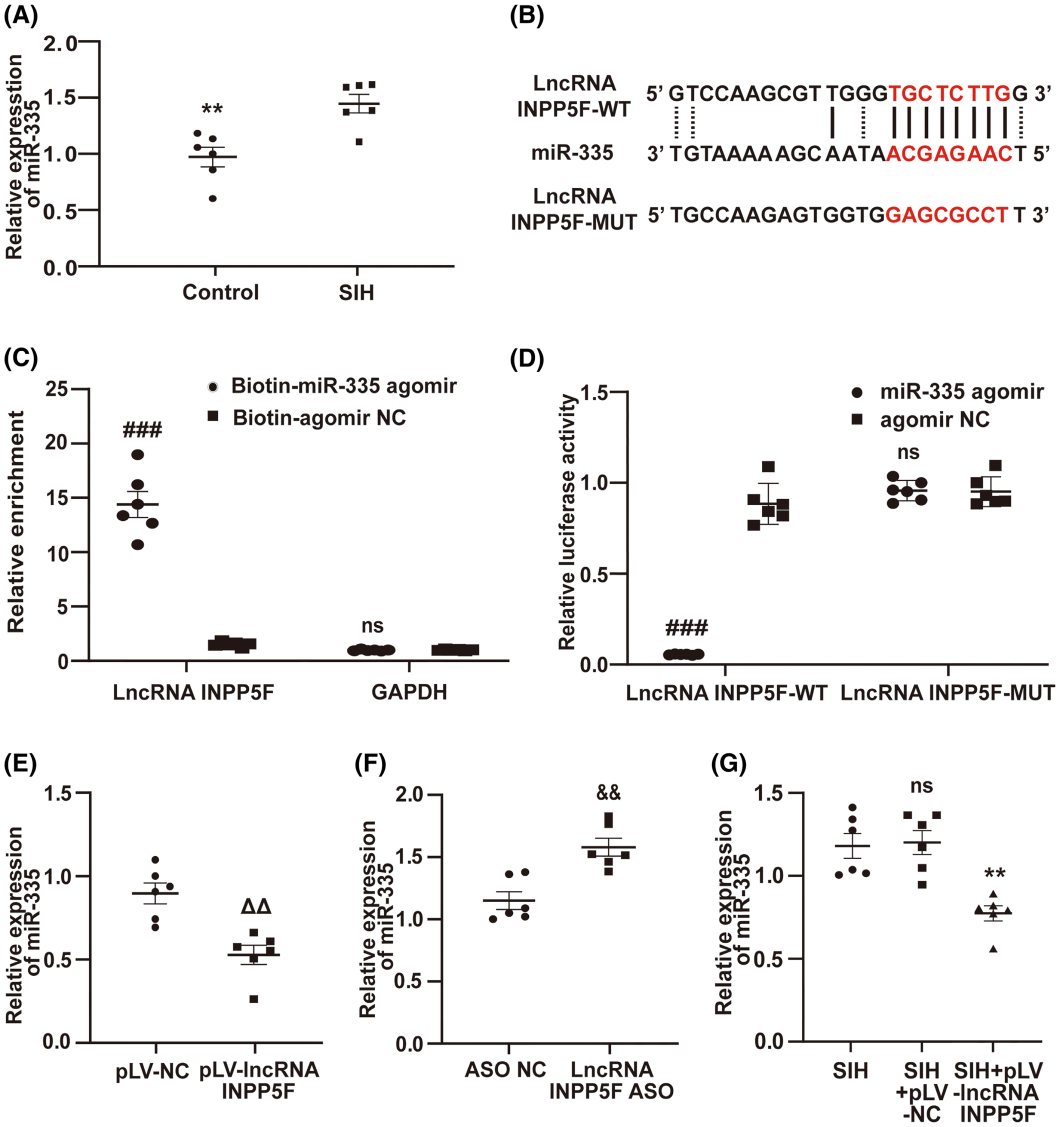
LncRNA INPP5F interacted with and negatively regulated miR‐335. (A) qRT‐PCR analysis of miR‐335 expression in RVLM of SIH and control rats. (B) Schematic of lncRNA INPP5F‐WT and lncRNA INPP5F‐MUT binding sites for miR‐335. (C) Biotinylated miR‐335 transfected into B104 cells with lncRNA INPP5F overexpression. LncRNA INPP5F expression was examined by qRT‐PCR after streptavidin capture. (D) Luciferase assay of B104 cells co‐transfected with lncRNA INPP5F‐WT reporter or lncRNA INPP5F‐MUT reporter and miR‐335 agomir or agomir NC. (E, F) qRT‐PCR analysis of miR‐335 expression in B104 cells with lncRNA INPP5F overexpression or silencing. (G) MiR‐335 expression was evaluated using qRT‐PCR after microinjection of pLV‐lncRNA INPP5F plasmid in RVLM of SIH rats. Data were presented as mean ± SEM. Statistical significance was determined by two‐tailed unpaired Student's *t*‐test (A, C–F) and one‐way ANOVA, followed by post hoc Bonferroni test (G). *n* = 6 rats per group (A, G). *n* = 6 of independent cell culture preparations (C–F). **p < 0.01, ns means non‐significant versus SIH group. ^###^ *p* < 0.001, ns means non‐significant versus agomir NC group. ^ΔΔ^ *p* < 0.01 versus pLV‐NC group. ^&&^ *p* < 0.01 versus ASO NC group. ASO, antisense oligonucleotide; MUT, mutant; NC, negative control; qRT‐PCR, quantitative reverse transcription polymerase chain reaction; RVLM, rostral ventrolateral medulla; SEM, standard error of the mean; SIH, stress‐induced hypertension; WT, wild type.

The underneath mechanism by which lncRNA INPP5F regulated Cttn expression involved in SIH progression continued to be unveiled. Accumulating evidence has confirmed that the lncRNA–miRNA–mRNA network may play a key role in many physiological and pathological processes.^45^ LncRNA INPP5F was mainly located in the cytoplasm (Figure 2E–G). This work hypothesized that lncRNA INPP5F could act as a competing endogenous miRNA sponge to regulate the expression of downstream Cttn gene. The authors' previous study suggested that downregulation of miR‐335 in RVLM exerted protective effects against neuronal apoptosis in SIH.^23^ qRT‐PCR indicated that the expression of miR‐335 was significantly higher in RVLM of SIH rats than that in control rats (Figure 6A). Figure 2C shows that lncRNA INPP5F was downregulated in RVLM of SIH rats. An inverse correlation between lncRNA INPP5F and miR‐335 expression was identified. The TargetScan and miRanda algorithms showed the binding sequence between lncRNA INPP5F and miR‐335 (Figure 6B). Biotin‐labeled miR‐335 was used to pull down lncRNA INPP5F in B104 cells with lncRNA INPP5F overexpression to further consolidate the direct binding of lncRNA INPP5F and miR‐335. The results demonstrated that lncRNA INPP5F was enriched in the group of biotinylated miR‐335 agomir (Figure 6C). Next, the results of luciferase reporter assay demonstrated that the luciferase activity of lncRNA INPP5F‐WT reporter, but not that of lncRNA INPP5F‐MUT reporter, was decreased by miR‐335 overexpression (Figure 6D). Besides, B104 cells were transfected with the pLV‐lncRNA INPP5F plasmid or LncRNA INPP5F ASO to disclose the effect of lncRNA INPP5F on miR‐335 expression. The overexpression efficiency of lncRNA INPP5F (Figure S6C) and the silencing efficiency of lncRNA INPP5F ASO (Figure S6B) in B104 cells were examined after 48 h of transfection. The results revealed that overexpression of lncRNA INPP5F decreased the expression of miR‐335 in B104 cells, and knockdown of lncRNA INPP5F increased it (Figure 6E,F). Their relationship was further confirmed in vivo, and the results indicated that the expression of miR‐335 in RVLM of SIH + pLV‐lncRNA INPP5F rats observably decreased compared with that in SIH and SIH + pLV‐NC rats (Figure 6G). The overexpression efficiency of lncRNA INPP5F in RVLM is illustrated in Figure S6A. Taken together, these data confirmed that lncRNA INPP5F served as a sponge for miR‐335.

### 3.5 MiR‐335 was associated with Cttn

**FIGURE 7:**
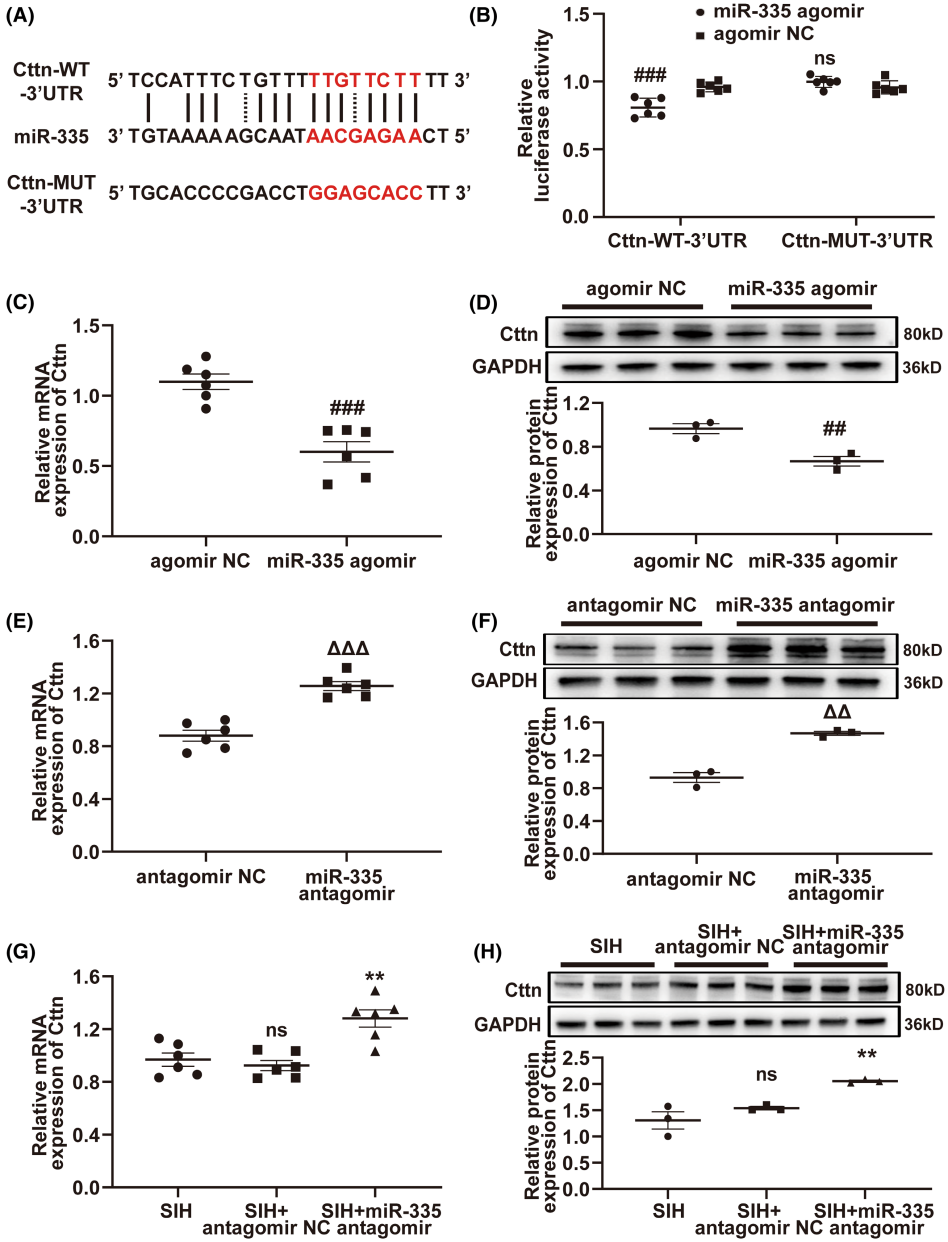
Cttn was a direct target of miR‐335. (A) Putative binding sites between miR‐335 and Cttn, as predicted by RNAhybrid software. (B) Relative luciferase activities of Cttn‐WT‐3′ UTR reporter and Cttn‐MUT‐3′ UTR reporter in B104 cells co‐transfected with miR‐335 agomir or agomir NC. (C–F) Effects of miR‐335 agomir and antagomir on Cttn expression, as determined by qRT‐PCR and Western blot analyses. (G, H) qRT‐PCR and Western blot experiments to test the expression of Cttn following miR‐335 inhibition in RVLM of SIH rats. Data were presented as mean ± SEM. Statistical significance was determined by two‐tailed unpaired Student's *t*‐test (B–F) and one‐way ANOVA, followed by post hoc Bonferroni test (G, H). *n* = 6 of independent cell culture preparations (B, C, E). *n* = 3 of independent cell culture preparations (D, F). *n* = 6 rats per group (G). *n* = 3 rats per group (H). ^##^ *p* < 0.01 and ^###^ *p* < 0.001, ns means non‐significant versus agomir NC group. ^ΔΔ^ *p* < 0.01 and ^ΔΔΔ^ *p* < 0.001 versus antagomir NC group. **p < 0.01, ns means non‐significant versus SIH group. MUT, mutant; NC, negative control; qRT‐PCR, quantitative reverse transcription polymerase chain reaction; RVLM, rostral ventrolateral medulla; SEM, standard error of the mean; SIH, stress‐induced hypertension; UTR, untranslated region; WT, wild type.

A series of experiments were performed to understand the relationship between miR‐335 and Cttn to further explore the in‐depth mechanisms of lncRNA INPP5F functioning as a ceRNA to modulate Cttn expression by binding miR‐335 participating in SIH development. The miR‐335 and Cttn expression profiles showed an opposite pattern in RVLM of SIH and control rats (Figure 6A; Figure 4A). The target prediction tool RNAhybrid revealed that Cttn had potential binding sites for miR‐335 (Figure 7A). B104 cells were co‐transfected with Cttn‐WT‐ or Cttn‐MUT‐3′ UTR reporter plasmid and miR‐335 agomir or agomir NC for dual‐luciferase reporter assay. The results revealed that after the overexpression of miR‐335, the luciferase activity of Cttn‐WT‐3′ UTR reporter was dramatically decreased while the luciferase activity of Cttn‐MUT‐3′ UTR reporter showed no obvious change (Figure 7B). Then, the effect of miR‐335 overexpression or miR‐335 knockdown on Cttn expression was probed. After the miR‐335 overexpression efficiency (Figure S6D) and miR‐335 knockdown efficiency (Figure S6E) in B104 cells were detected, qRT‐PCR and Western blot were used to examine the expression level of Cttn. Upregulation of miR‐335 caused downregulation of Cttn in B104 cells (Figure 7C,D). By contrast, knockdown of miR‐335 showed an opposite change (Figure 7E,F). Further in vivo study proved that the expression of Cttn was markedly increased in RVLM of SIH + antagomir rats compared with the SIH and SIH + antagomir NC rats (Figure 7G,H). Figure S6F demonstrates the inhibition efficiency of miR‐335 antagomir in RVLM. These results provided evidence that miR‐335 targeted Cttn.

### 3.6 LncRNA INPP5F regulated Cttn expression by sponging miR‐335

**FIGURE 8:**
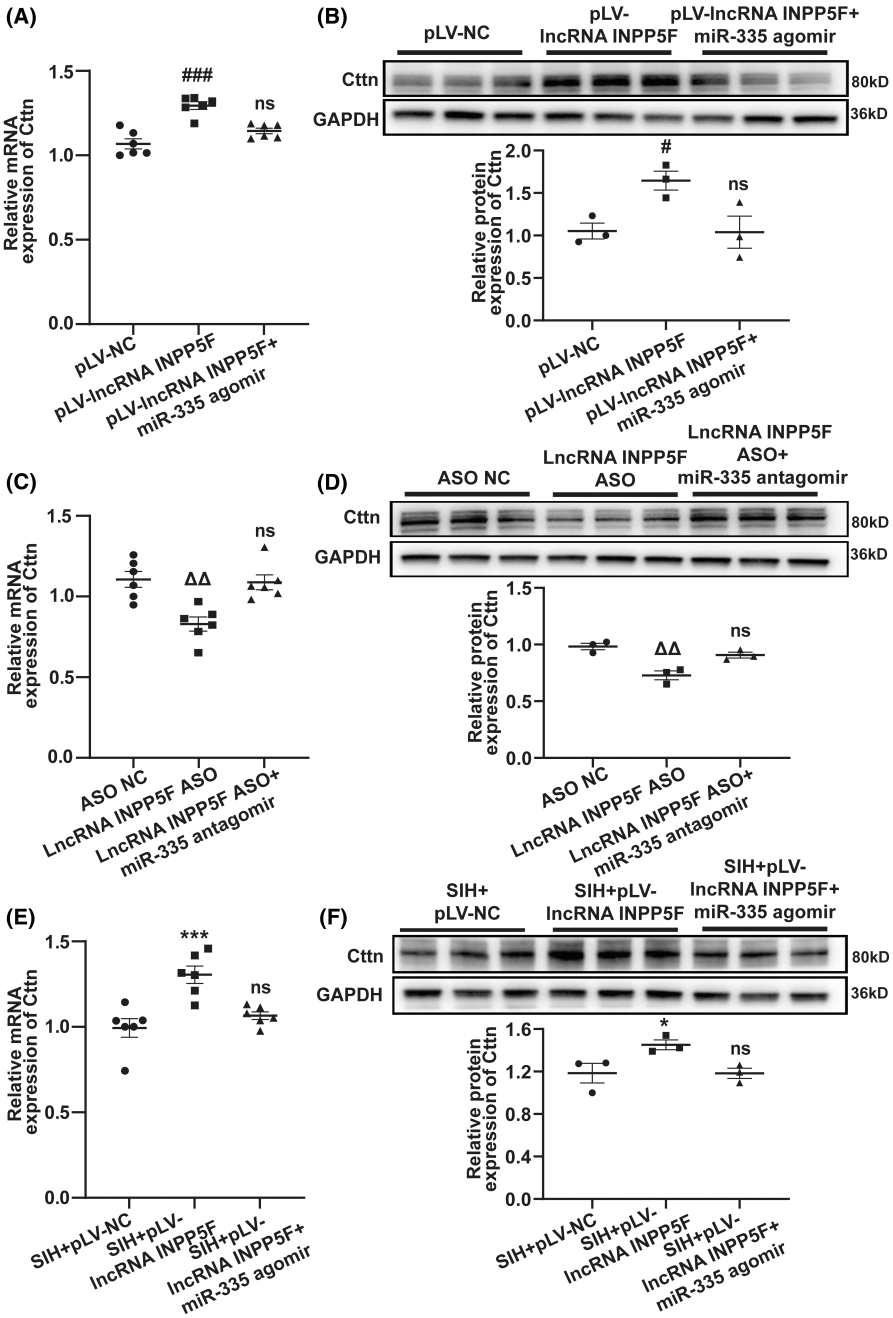
LncRNA INPP5F modulated Cttn expression by absorbing miR‐335. (A–D) qRT‐PCR and Western blot analyses of the effects of lncRNA INPP5F overexpression and miR‐335 agomir or lncRNA INPP5F silencing and miR‐335 antagomir on the mRNA and protein levels of Cttn in B104 cells. (E, F) The mRNA and protein expression levels of Cttn were detected by qRT‐PCR and Western blot in SIH rats after microinjection of pLV‐lncRNA INPP5F plasmid and miR‐335 agomir. Data were presented as mean ± SEM. Statistical significance was determined by one‐way ANOVA, followed by post hoc Bonferroni test (A–F). *n* = 6 of independent cell culture preparations (A, C). *n* = 3 of independent cell culture preparations (B, D). *n* = 6 rats per group (E). *n* = 3 rats per group (F). ^#^ *p* < 0.05, ^###^ *p* < 0.001, ns means non‐significant versus pLV‐NC group. ^ΔΔ^ *p* < 0.01, ns means non‐significant versus ASO NC group. *p < 0.05, ***p < 0.001, ns means non‐significant versus SIH+pLV‐NC group. ASO, antisense oligonucleotide; NC, negative control; qRT‐PCR, quantitative reverse transcription polymerase chain reaction; SEM, standard error of the mean; SIH, stress‐induced hypertension.

As mentioned, lncRNA INPP5F was a sponge of miR‐335, which targeted Cttn. Thus, whether lncRNA INPP5F modulated Cttn expression by sponging miR‐335 was further examined. As shown in Figure 8A,B, overexpression of lncRNA INPP5F remarkably increased the expression of Cttn in B104 cells. MiR‐335 agomir could reverse the promotion effect of lncRNA INPP5F on Cttn expression. The mRNA and protein levels of Cttn were markedly downregulated by lncRNA INPP5F knockdown in B104 cells, and miR‐335 antagomir attenuated these effects (Figure 8C,D). In the in vivo experiment, qRT‐PCR and Western blot assays indicated that in RVLM of SIH + pLV‐lncRNA INPP5F rats, the mRNA and protein expression levels of Cttn were dramatically elevated, whereas miR‐335 agomir treatment significantly reduced the Cttn expression (Figure 8E,F). Collectively, lncRNA INPP5F could regulate Cttn expression via sponging miR‐335.

## 4 DISCUSSION

Accompanied by the rapid promotion of high‐throughput sequencing, it became evident that eukaryotic genome transcribes up to 90% of the genomic DNA, and 98% of these transcripts are transcribed as ncRNAs.^46^ LncRNAs are a relatively well‐characterized class of ncRNA molecules, and they have been detected in different species. Owing to their ubiquitous existence, lncRNAs have been widely investigated and confirmed to be involved in the regulation of multiple pathological processes, such as hypertension.^20^, ^21^, ^22^ However, very little research has been conducted on the relation of lncRNAs to the neurogenic characteristics of hypertension. RVLM is a critical region that is responsible for the regulation of sympathetic outflow and hypertension.^47^ Here, a comprehensive picture of the dynamic RVLM lncRNA transcriptome changes taking part in the pathological course of SIH was presented, further focusing on the function of these lncRNAs to understand their detailed regulatory mechanisms in SIH at the molecular level. These valuable lncRNAs may lead to potential opportunities for identifying novel therapeutic targets of SIH.

Normally, expression alteration is important to illustrate biological differences in many different physiological or pathological states and critical for obtaining potential therapeutic targets and diagnostic biomarkers. Computational analysis data revealed 39 dysregulated lncRNAs between the control and SIH groups. Not all these 39 dysregulated lncRNAs may have functions in SIH; some could be the biological differences between samples or produce a response and compensation. Among them, the lncRNA INPP5F expression in SIH rats was significantly downregulated compared with that in control rats, as further confirmed by qRT‐PCR. LncRNA INPP5F has five exons, and it was derived from Inpp5f gene. In vivo means “within the living” in Latin, and it refers to experimentation utilizing a whole living organism, with a high degree of accuracy and translatability. It is crucial in function validation and novel therapy development. Gain‐of‐function experiments demonstrated that the lncRNA INPP5F overexpression in RVLM notably lowered the SBP, MAP, HR, RSNA, plasma NE, and the number of RVLM c‐Fos‐positive TH+ neurons in SIH rats. Therefore, the stress‐induced dysregulation of lncRNA INPP5F in RVLM could modulate the blood pressure, sympathetic nerve activity, and neuronal excitability involved in the pathogenesis of SIH.

The apoptosis of RVLM neurons promotes sympathetic outflow and accelerates the chances of hypertension.^25^, ^37^ Cortactin (CTTN), which is encoded by Cttn, exerts a key function in coupling membrane dynamics to cortical actin assembly^48^ and has an anti‐apoptotic effect in various diseases.^38^, ^39^, ^49^ The PI3K‐Akt signaling pathway modulates diverse cellular processes, including cell apoptosis.^40^, ^41^, ^50^ Cttn is considered as an upstream of the PI3K‐AKT pathway,^42^, ^43^ which is related to apoptosis.^43^, ^44^ In the present study, lncRNA INPP5F overexpression significantly increased the expression of Cttn, p‐PI3K, and p‐AKT, whereas lncRNA INPP5F knockdown markedly reduced their expression levels. Furthermore, a series of in vivo and in vitro experiments illustrated that lncRNA INPP5F inhibited neuronal apoptosis via activating the Cttn/PI3K‐AKT axis to impede SIH progression. Considering this conclusion, the molecular mechanisms underlying the effect of lncRNA INPP5F on Cttn expression were explored.

LncRNAs are known to modulate gene expression and exert biological functions in several modes, such as affecting the chromatin structure and regulating promoters and enhancers.^51^, ^52^ An increasing body of evidence suggested that lncRNAs harbor miRNA recognition elements and thus compete with mRNAs to bind miRNAs, which may exert critical effects on many different diseases. Li et al.^53^ summarized that lnc‐APUE regulated cell‐cycle progression and tumor growth via the miR‐20b/E2F1 axis. Lnc‐ISG20 controlled NFAT5 expression by sponging miR‐486‐5p in diabetic nephropathy.^54^ The lncRNA–miRNA–mRNA networks have also contributed to many other illnesses, such as Alzheimer's disease and heart failure.^55^, ^56^ LncRNA subcellular localization provides valuable clues to discover their molecular function. LncRNAs situated in the cytoplasm can act as miRNA sponges that influence the expression of related downstream target genes.^36^, ^57^ Based on the bioinformatics prediction and FISH and qRT‐PCR results, lncRNA INPP5F was predominately distributed in the cytoplasm, demonstrating that it may function as a ceRNA. MiR‐335 is highly conserved across humans, mice, and rats. Increasing evidence identified miR‐335 as a key regulator of a number of pathological processes. MiR‐335 has a huge effect on tumorigenesis and tumor progression. Wang et al.^58^ proposed that miR‐335 regulated cell cycle and metastasis in lung adenocarcinoma. MiR‐335 inhibited gastric cancer progression through regulating the level of Mapk10.^59^ MiR‐335, which was remarkably downregulated in breast cancer, suppressed tumor growth by targeting Sdc1.^60^ Moreover, it was also implicated in many other grave illnesses, such as type 2 diabetes, osteoarthritis, and polycystic ovary syndrome.^61^, ^62^, ^63^ The authors' previous study proved that miR‐335 could serve as an apoptosis enhancer by inhibiting Sphk1 to increase the sympathetic vasoconstriction involved in SIH.^25^ The present work robustly proved that lncRNA INPP5F has the ability to capture miR‐335 through biotinylated RNA pulldown, dual‐luciferase reporter, and gain‐ and loss‐of‐function assays. Whether Cttn was a downstream gene of miR‐355 was then explored. Dual‐luciferase reporter and gain‐ and loss‐of‐function assays confirmed that Cttn was a direct target of miR‐335. The relationship between lncRNA INPP5F and Cttn was further elucidated. In vitro and vivo data revealed that lncRNA INPP5F upregulation increased Cttn expression, whereas lncRNA INPP5F downregulation decreased it. However, miR‐335 agomir or antagomir treatment impaired these effects. Such findings implied that lncRNA INPP5F could modulate Cttn expression via sponging miR‐335.

To the best of the authors' knowledge, this research is the first to provide systematic insights into functional lncRNA signatures in RVLM participating in central regulation of SIH. LncRNA INPP5F repressed SIH progression via regulating RVLM neuronal apoptosis, and the underlying mechanism was that lncRNA INPP5F induced Cttn expression via absorbing miR‐335. Notably, this study has some limitations. First, female rats were not used. However, previous research indicated that the sex difference was associated with the pathogenesis of hypertension.^64^ Next, other target genes and signals in RVLM engaging in lncRNA INPP5F‐regulated SIH progression remain to get further research. Last, other central sites, such as PVN, NTS, and CVLM, play key roles in controlling sympathetic tone and blood pressure. The changes in lncRNA INPP5F expression in these tissues and the potential molecular mechanisms should also be studied. These efforts would be great challenges in the upcoming years. Moreover, in our preliminary experiments, qPCR analysis demonstrated that lncRNA INPP5F did not show differential expression in RVLM of spontaneously hypertensive rats and dahl salt‐sensitive rats compared with controls (data not shown). The change in lncRNA INPP5F expression in RVLM was specially induced by stress and further affected sympathetic outflow, which participated in SIH progression.

Chronic stress can be perceived by the brain and increases the risk for SIH. Stress induces RVLM neuronal excitation and further causes sympathetic hyperactivity, which seems to be the major factor causing SIH. The rat model, which is established by long‐term continuous plantar stimulation and noise exposure, is considered the reliable model for investigating the progression of SIH.^13^, ^14^, ^15^, ^16^, ^17^ This study indicated that the decreased expression of lncRNA INPP5F in RVLM is a common event in SIH rats. Elevating lncRNA INPP5F expression markedly ameliorated the stress‐induced increase in blood pressure, sympathetic nerve activity, and neuronal excitability. Mechanistically, lncRNA INPP5F interacted with miR‐335 and promoted the expression of Cttn, thereby activating the PI3K‐AKT/anti‐apoptosis axis to suppress the progression of SIH. The data elucidated that the expression changes in RVLM lncRNAs could regulate the sympathetic outflow, which was involved in SIH pathogenesis. Targeting lncRNA INPP5F may serve as an effective strategy for the treatment of SIH.

## AUTHOR CONTRIBUTIONS

S.Z. and D.S.D. conceived and designed research. S.Z., G.J.C., X.P.W., L.T., L.P.W., T.F.L., L.C.Z., S.M.Z., and H.S.L. performed the experiments. S.Z., G.J.C., and X.P.W. analyzed and interpreted the data. S.Z. wrote the manuscript, which was read, edited, and approved by all the authors. S.Z. and D.D.S. contributed reagents, materials, and analysis tools.

## FUNDING INFORMATION

This work was supported by the National Natural Science Foundation of China (32071111, 31871151, 31571171, and 32200929) and the Natural Science Foundation of Shandong Province (ZR202112030301).

## CONFLICT OF INTEREST STATEMENT

The authors have declared that no conflict of interest exists.

## Supporting information


Appendix S1

## Data Availability

The data that support the findings of this study are available from the corresponding author upon reasonable request. The lncRNA‐sequencing clean data reported in this study have been deposited in the NCBI Sequence Read Archive (SRA). The accession number is PRJNA930747.
