# Supplementary material for: LncRNA INPP5F ameliorates stress‐induced hypertension via the miR‐335/Cttn axis in rostral ventrolateral medulla
Source: CNS Neurosci Ther. 2023 Feb 27;29(7):1830–47. doi: 10.1111/cns.14142 (PMC10324364; doi:10.1111/cns.14142)
Supplement: Supplementary file 1 — Appendix S1 [file CNS-29-1830-s001.pdf]

Supplementary Figures

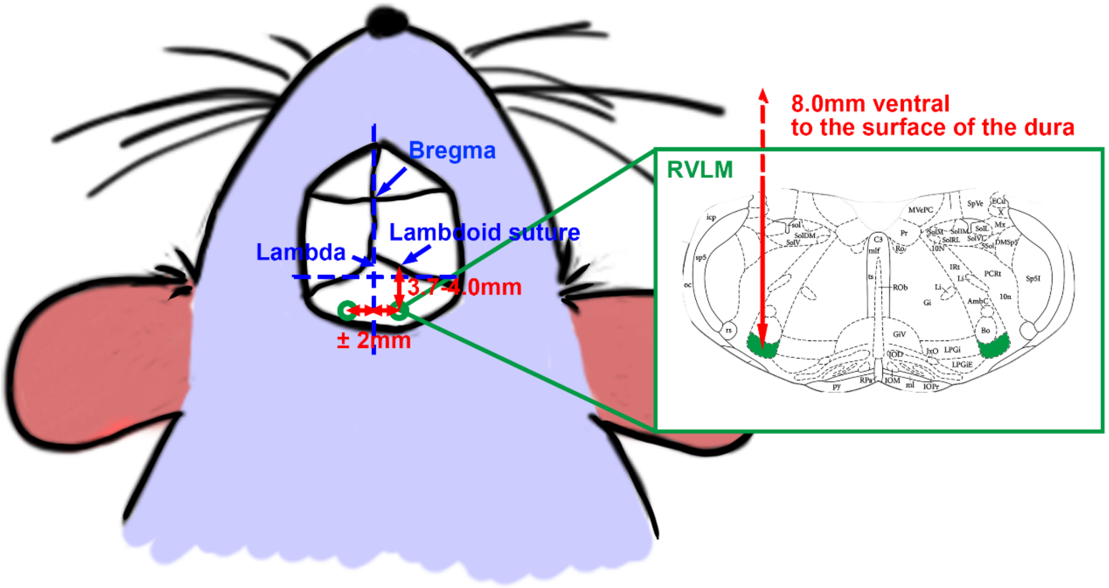

Figure S1 The diagram which was used to demonstrate the process of confirming the correct positioning of the RVLM.

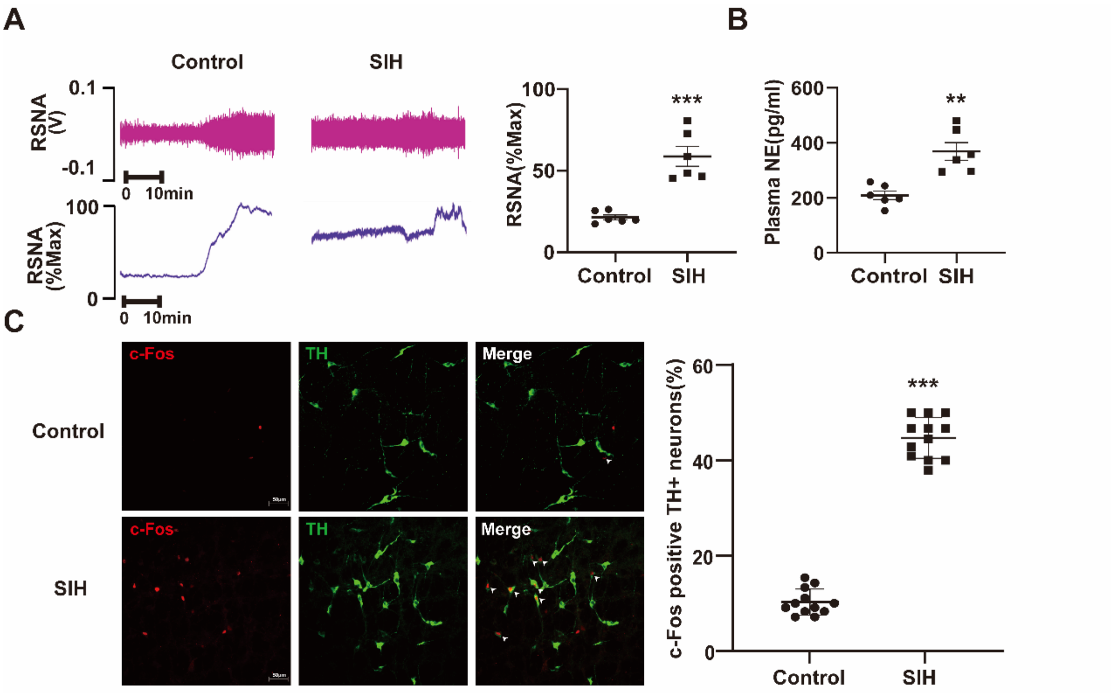

Figure S2 (**A–C**) Chronic stress increased RSNA, plasma NE and c-Fos-positive TH+ neurons in rats. Data were presented as mean ± SEM. Statistical significance was determined by two-tailed unpaired Student's *t*-test (**A–C**). n = 6 rats per group (**A, B**). n = 12 slices from 6 rats, two slices per rat (**C**). $^{**}p < 0.01$, $^{***}p < 0.001$ versus control group. Abbreviations: NE, norepinephrine; RSNA, renal sympathetic nerve activity; SEM, standard error of the mean; SIH, stress-induced hypertension; TH, tyrosine hydroxylase.

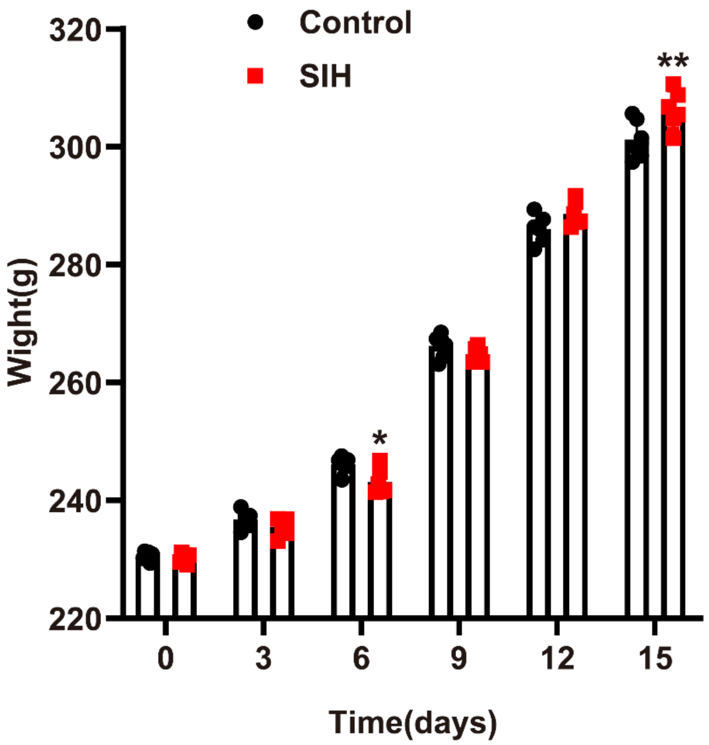

Figure S3 Variability in body weight between SIH and control rats. Data were presented as mean ± SEM. Statistical significance was determined by two-tailed unpaired Student's *t*-test. n = 6 rats per group. $^{*}p < 0.05$, $^{**}p < 0.01$ versus control group. Abbreviations: SEM, standard error of the mean; SIH, stress-induced hypertension.

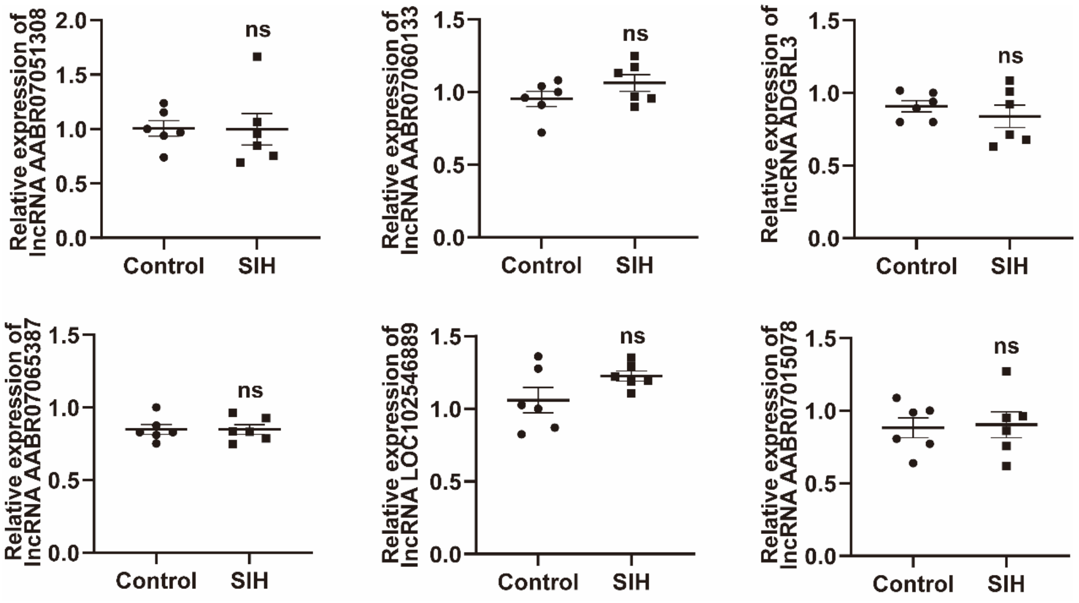

Figure S4 Relative expression of the 6 indicated lncRNAs listed in Figure 2B measured by qRT-PCR. Data were presented as mean ± SEM. Statistical significance was determined by two-tailed unpaired Student's *t*-test. n = 6 rats per group. ns means nonsignificant versus control group. Abbreviations: qRT-PCR, quantitative reverse transcription polymerase chain reaction; SEM, standard error of the mean; SIH, stress-induced hypertension.

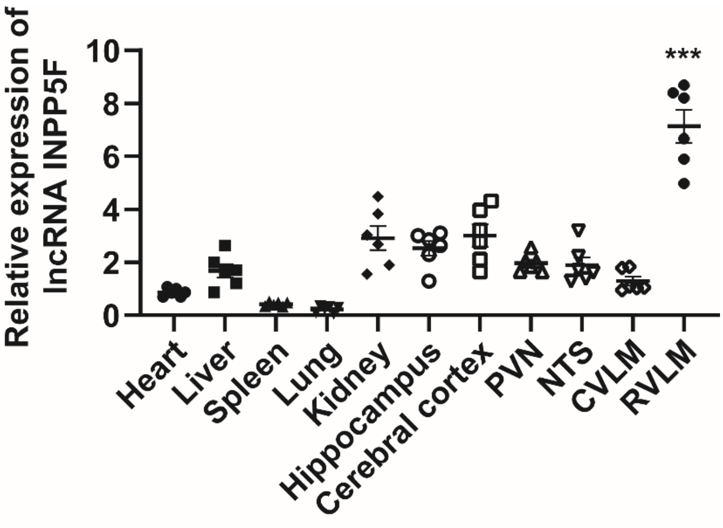

Figure S5 Relative expression of lncRNA INPP5F in 11 tissues of control rats measured by qRT-PCR. Data were presented as mean ± SEM. Statistical significance was determined by two-tailed unpaired Student's *t*-test. n = 6 rats per group. $^{***}p < 0.001$ versus heart group. Abbreviations: qRT-PCR, quantitative reverse transcription polymerase chain reaction; SEM, standard error of the mean.

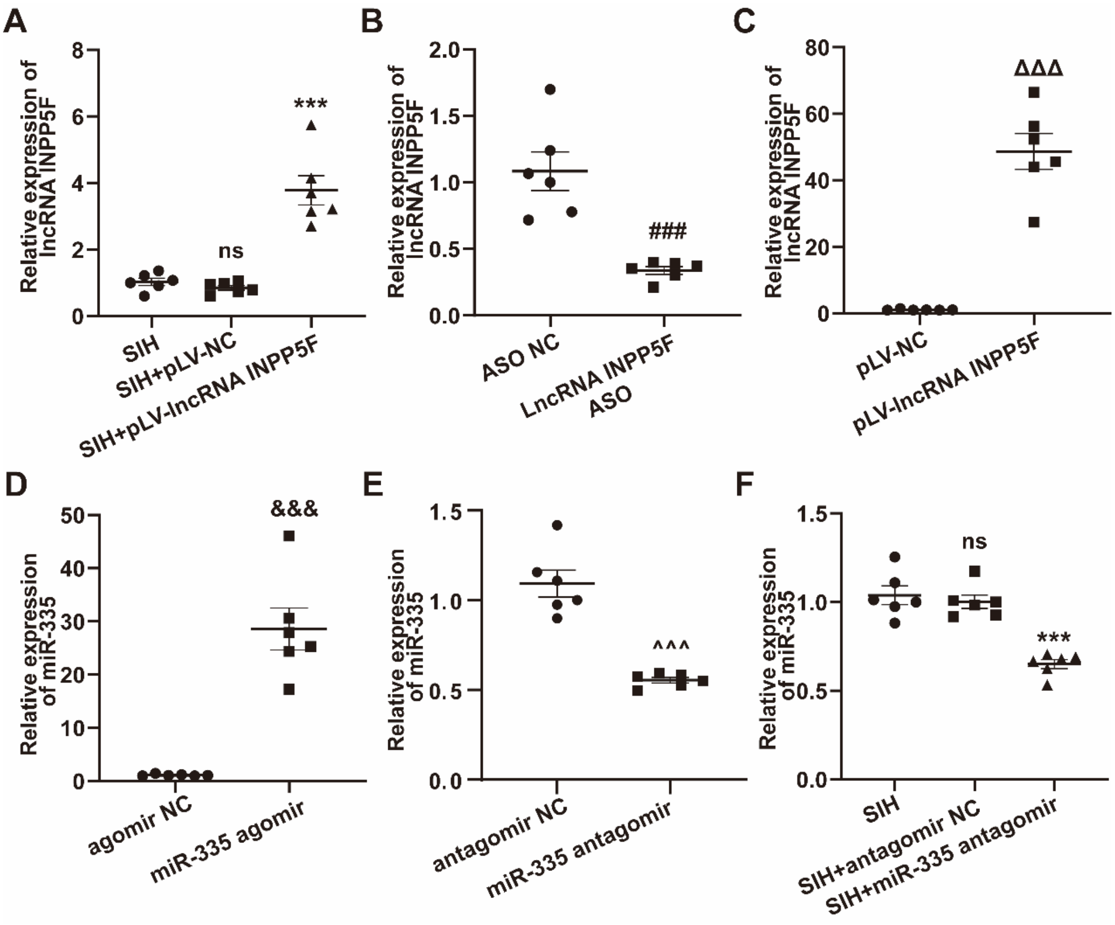

Figure S6 (**A**) lncRNA INPP5F overexpression efficiency in RVLM, as determined by qRT-PCR. (**B**) qRT-PCR analysis of lncRNA INPP5F expression in B104 cells after being transfected with lncRNA INPP5F ASO. (**C**) Overexpression efficiency of lncRNA INPP5F, as determined by qRT-PCR, in B104 cells. (**D, E**) qRT-PCR assay to assess the overexpression and knockdown efficiency

of miR-335 in B104 cells. (**F**) Evaluation of miR-335 silencing efficiency in RVLM by qRT-PCR. Data were presented as mean ± SEM. Statistical significance was determined by two-tailed unpaired Student's *t*-test (**B–E**) and one-way ANOVA, followed by post-hoc Bonferroni test (**A, F**). n = 6 rats per group (**A, F**). n=6 of independent cell culture preparations (**B–E**). $^{***}p < 0.001$, ns means nonsignificant versus SIH group. $^{###}p < 0.001$ versus ASO NC group. $^{\Delta\Delta\Delta}p < 0.001$ versus pLV-NC group. $^{\&\&\&}p < 0.001$ versus agomir NC group. $^{\wedge\wedge\wedge}p < 0.001$, versus antagomir NC group. Abbreviations: ASO, antisense oligonucleotide; NC, negative control; qRT-PCR, quantitative reverse transcription polymerase chain reaction; RVLM, rostral ventrolateral medulla; SEM, standard error of the mean; SIH, stress-induced hypertension.

Supplementary Tables

Table S1 LncRNA INPP5F ASO, miR-335 agomir, agomir NC, miR-335 antagomir, antagomir NC used for intra-RVLM microinjection and cell transfection

Acession No.	Sequence (5'-3')
LncRNA INPP5F ASO	CTTCAGAAGGCTAGTGATGG
miR-335 agomir	UCAAGAGCAAUAACGAAAAAUGU
	AUUUUUCGUUAUUGCUCUUGAUU
agomir NC	UUUAAAGAACGUCUCAGGUUU
	ACGUGACACGUUCGGAGAAUU
miR-335 antagomir	ACAUUUUUCGUUAUUGCUCUUGA
antagomir NC	CAGUACUUUUGUGUAGUACAA

Abbreviations: ASO, antisense oligonucleotide; NC, negative control; RVLM, rostral ventrolateral medulla.

Table S2 The full statistical reports.

item	Degrees of freedom (df)	*t/F* value	*P* value
Figure 2A			
	Degrees of freedom (df)	*t* value	*P* value
Systolic blood pressure (SBP) before stress	10	0.7545	0.4686
Systolic blood pressure (SBP) on the 5th day after stress	10	3.019	0.0129
Systolic blood pressure (SBP) on the 10th day after stress	10	14.47	<0.0001
Systolic blood pressure (SBP) on the 15th day after stress	10	10.36	<0.0001
Mean arterial pressure (MAP) before stress	10	0.05631	0.9562
Mean arterial pressure (MAP) on the 5th day after stress	10	2.854	0.0171
Mean arterial pressure (MAP) on the 10th day after stress	10	9.358	<0.0001
Mean arterial pressure (MAP) on the 15th day after stress	10	21.1	<0.0001
Heart rate (HR) before stress	10	0.008695	0.9324
Heart rate (HR) on the 5th day after stress	10	0.5905	0.5679
Heart rate (HR) on the 10th day after stress	10	4.29	0.0016
Heart rate (HR) on the 15th day after stress	10	6.969	<0.0001
Figure 2C			
	Degrees of freedom (df)	*t* value	*P* value
Relative expression of lncRNA INPP5F	10	6.399	<0.0001
Relative expression of lncRNA ST3GAL4	10	3.361	0.0072

Relative expression of lncRNA WAP1	10	2.545	0.0291
Relative expression of lncRNA AABR07068852	10	3.09	0.0114

Figure 2D

	Degrees of freedom (df)	*F* value	*P* value
Relative expression of lncRNA INPP5F	df1=10; df2=55	63.23	<0.0001

Figure 3A

	Degrees of freedom (df)	*F* value	*P* value
Systolic blood pressure (SBP) before stress	df1=2; df2=15	0.4578	0.6412
Systolic blood pressure (SBP) on the 5th day after stress	df1=2; df2=15	10.03	0.0017
Systolic blood pressure (SBP) on the 10th day after stress	df1=2; df2=15	84.31	<0.0001
Systolic blood pressure (SBP) on the 15th day after stress	df1=2; df2=15	22.69	<0.0001
Mean arterial pressure (MAP) before stress	df1=2; df2=15	0.2223	0.8032
Mean arterial pressure (MAP) on the 5th day after stress	df1=2; df2=15	9.57	0.0021
Mean arterial pressure (MAP) on the 10th day after stress	df1=2; df2=15	68.92	<0.0001
Mean arterial pressure (MAP) on the 15th day after stress	df1=2; df2=15	34.87	<0.0001
Heart rate (HR) before stress	df1=2; df2=15	0.08432	0.9196
Heart rate (HR) on the 5th day after stress	df1=2; df2=15	0.3066	0.7404
Heart rate (HR) on the 10th day after stress	df1=2; df2=15	51.4	<0.0001
Heart rate (HR) on the 15th day after stress	df1=2; df2=15	33.83	<0.0001

Figure 3B

	Degrees of freedom (df)	*F* value	*P* value
Renal sympathetic nerve activity (RSNA, %Max)	df1=2; df2=15	8.998	0.0027

Figure 3C

	Degrees of freedom (df)	*F* value	*P* value
Plasma norepinephrine (NE)	df1=2; df2=15	9.575	0.0021

Figure 3D

	Degrees of freedom (df)	*F* value	*P* value
c-Fos-positive TH+ neurons (%)	df1=2; df2=33	29.03	<0.0001

Figure 4A

	Degrees of freedom (df)	*t* value	*P* value
Relative protein expression of Cttn	4	7.648	0.0016
Relative protein expression of p-PI3K	4	2.934	0.0427
Relative protein expression of p-AKT	4	3.264	0.031

Figure 4B

	Degrees of freedom (df)	*F* value	*P* value
Relative protein expression of Cttn	df1=2; df2=6	15.45	0.0043
Relative protein expression of p-PI3K	df1=2; df2=6	33.13	0.0006
Relative protein expression of p-AKT	df1=2; df2=6	12.28	0.0076
Relative protein expression of BCL2	df1=2; df2=6	12.99	0.0066
Relative protein expression of BAX	df1=2; df2=6	33.88	0.0005
Relative protein expression of cleaved Caspase 3	df1=2; df2=6	96.06	<0.0001

Figure 4C

	Degrees of freedom (df)	*F* value	*P* value
Cleaved caspase 3-positive neural cells (%)	df1=2; df2=33	51.76	<0.0001

Figure 4D

	Degrees of freedom (df)	*F* value	*P* value
TUNEL-positive neural cells (%)	df1=2; df2=33	195.5	<0.0001

Figure 5A

	Degrees of freedom (df)	*t* value	*P* value
Relative protein expression of Cttn	4	6.25	0.0033
Relative protein expression of p-PI3K	4	8.256	0.0012
Relative protein expression of p-AKT	4	6.857	0.0024
Relative protein expression of BCL2	4	4.348	0.0122
Relative protein expression of BAX	4	6.784	0.0025
Relative protein expression of cleaved Caspase 3	4	3.398	0.0273

Figure 5B

	Degrees of freedom (df)	*t* value	*P* value
Cleaved caspase 3-positive cells (%)	22	13.12	<0.0001

Figure 5C

	Degrees of freedom (df)	*t* value	*P* value
TUNEL-positive cells (%)	22	23.36	<0.0001

Figure 5D

	Degrees of freedom (df)	*t* value	*P* value
Cell viability (%)	22	12.41	<0.0001

Figure 5E

	Degrees of freedom (df)	*t* value	*P* value
48h cell viability (%)	10	8.735	<0.0001
72h cell viability (%)	10	13.7	<0.0001
96h cell viability (%)	10	15.36	<0.0001

Figure 5F

	Degrees of freedom (df)	*t* value	*P* value
Apoptosis cells (%)	10	11.58	<0.0001

Figure 6A

	Degrees of freedom (df)	*t* value	*P* value
Relative expression of miR-335	10	3.961	0.0027

Figure 6C

	Degrees of freedom (df)	*t* value	*P* value
Relative enrichment of lncRNA INPP5F	10	10.84	<0.0001
Relative enrichment of GAPDH	10	0.6728	0.5163

Figure 6D

	Degrees of freedom (df)	*t* value	*P* value
Relative luciferase activity of lncRNA INPP5F-WT	10	18.12	<0.0001
Relative luciferase activity of lncRNA INPP5F-MUT	10	0.1307	0.8986

Figure 6E

	Degrees of freedom (df)	*t* value	*P* value
Relative expression of miR-335	10	4.339	0.0015

Figure 6F

	Degrees of freedom (df)	*t* value	*P* value

Relative expression of miR-335	10	4.247	0.0017
Figure 6G			
	Degrees of freedom (df)	*F* value	*P* value
Relative expression of miR-335	df1=2; df2=15	13.57	0.0004
Figure 7B			
	Degrees of freedom (df)	*t* value	*P* value
Relative luciferase activity of Cttn-WT-3’ UTR	10	4.817	0.0007
Relative luciferase activity of Cttn-MUT-3’ UTR	10	1.55	0.1521
Figure 7C			
	Degrees of freedom (df)	*t* value	*P* value
Relative mRNA expression of Cttn	10	5.517	0.0003
Figure 7D			
	Degrees of freedom (df)	*t* value	*P* value
Relative protein expression of Cttn	4	4.743	0.009
Figure 7E			
	Degrees of freedom (df)	*t* value	*P* value
Relative mRNA expression of Cttn	10	7.032	<0.0001
Figure 7F			
	Degrees of freedom (df)	*t* value	*P* value
Relative protein expression of Cttn	4	8.429	0.0011
Figure 7G			
	Degrees of freedom (df)	*F* value	*P* value
Relative mRNA expression of Cttn	df1=2; df2=15	13.55	0.0004
Figure 7H			
	Degrees of freedom (df)	*F* value	*P* value
Relative protein expression of Cttn	df1=2; df2=6	15.05	0.0046
Figure 8A			
	Degrees of freedom (df)	*F* value	*P* value
Relative mRNA expression of Cttn	df1=2; df2=15	23.59	<0.0001
Figure 8B			
	Degrees of freedom (df)	*F* value	*P* value
Relative protein expression of Cttn	df1=2; df2=6	6.342	0.0331
Figure 8C			
	Degrees of freedom (df)	*F* value	*P* value
Relative mRNA expression of Cttn	df1=2; df2=15	10.99	0.0011
Figure 8D			
	Degrees of freedom (df)	*F* value	*P* value
Relative protein expression of Cttn	df1=2; df2=6	17.31	0.0032
Figure 8E			
	Degrees of freedom (df)	*F* value	*P* value
Relative mRNA expression of Cttn	df1=2; df2=15	13.18	0.0005
Figure 8F			
	Degrees of freedom (df)	*F* value	*P* value
Relative protein expression of Cttn	df1=2; df2=6	5.523	0.0436

Figure S2A

	Degrees of freedom (df)	*t* value	*P* value
Renal sympathetic nerve activity (RSNA, %Max)	10	5.97	0.0001

Figure S2B

	Degrees of freedom (df)	*t* value	*P* value
Plasma norepinephrine (NE)	10	4.516	0.0011

Figure S2C

	Degrees of freedom (df)	*t* value	*P* value
c-Fos-positive TH+ neurons (%)	22	23.37	<0.0001

Figure S3

	Degrees of freedom (df)	*t* value	*P* value
weight before stress	10	0.6395	0.5369
weight on the third day after stress	10	1.533	0.1564
weight on the 6th day after stress	10	2.798	0.0189
weight on the 9th day after stress	10	1.621	0.136
weight on the 12th day after stress	10	1.959	0.0785
weight on the 15th day after stress	10	3.305	0.0079

Figure S4

	Degrees of freedom (df)	*t* value	*P* value
Relative expression of lncRNA AABR07051308	10	0.05925	0.9539
Relative expression of lncRNA AABR07060133	10	1.417	0.1869
Relative expression of lncRNA ADGRL3	10	0.7972	0.4439
Relative expression of lncRNA AABR07065387	10	0.02457	0.9809
Relative expression of lncRNA LOC102546889	10	1.746	0.1115
Relative expression of lncRNA AABR07015078	10	0.1856	0.8565

Figure S5

	Degrees of freedom (df)	*t* value	*P* value
Relative expression of lncRNA INPP5F	df1=10; df2=55	37.43	<0.0001

Figure S6A

	Degrees of freedom (df)	*F* value	*P* value
Relative expression of lncRNA INPP5F	df1=2; df2=15	38.26	<0.0001

Figure S6B

	Degrees of freedom (df)	*t* value	*P* value
Relative expression of lncRNA INPP5F	10	5.013	0.0005

Figure S6C

	Degrees of freedom (df)	*t* value	*P* value
Relative expression of lncRNA INPP5F	10	8.858	<0.0001

Figure S6D

	Degrees of freedom (df)	*t* value	*P* value
Relative expression of miR-335	10	6.961	<0.0001

Figure S6E

	Degrees of freedom (df)	*t* value	*P* value
Relative expression of miR-335	10	6.999	<0.0001

Figure S6F

	Degrees of freedom (df)	F value	P value
Relative expression of miR-335	df1=2; df2=15	28.5	<0.0001

Abbreviations: MUT, mutant; TH, tyrosine hydroxylase; TUNEL, TdT-mediated dUTP-biotin nick end labeling; WT, wild-type.
